# Inter-annual variability of land surface fluxes across vineyards: the role of climate, phenology, and irrigation management

**DOI:** 10.1007/s00271-022-00784-0

**Published:** 2022-04-15

**Authors:** N. Bambach, W. Kustas, J. Alfieri, F. Gao, J. Prueger, L. Hipps, L. McKee, S. J. Castro, M. M. Alsina, A. J. McElrone

**Affiliations:** 1 Department of Land, Air and Water Resources, University of California, Davis, Davis, CA 95616 USA; 2 Hydrology and Remote Sensing Laboratory, USDA-ARS, Beltsville, MD USA; 3 National Laboratory for Agriculture and the Environment, USDA-ARS, Ames, IA 50011 USA; 4 Department of Plants, Soils, and Climate, Utah State University, Logan, UT USA; 5 Department of Viticulture and Enology, University of California, Davis, Davis, CA 95616 USA; 6 E & J Gallo Winery, Viticulture, Chemistry and Enology, Modesto, CA USA; 7 Crops Pathology and Genetics Research Unit, USDA-ARS, Davis, CA 95616 USA

## Abstract

Irrigation and other agricultural management practices play a key role in land surface fluxes and their interactions with atmospheric processes. California’s Central Valley agricultural productivity is strongly linked to water availability associated with conveyance infrastructure and groundwater, but greater scrutiny over agricultural water use requires better practices particularly during extended and severe drought conditions. The future of irrigated agriculture in California is expected to be characterized neither by perpetual scarcity nor by widespread abundance. Thus, further advancing irrigation technologies and improving management practices will be key for California’s agriculture sustainability. In this study, we present micrometeorological observations from the Grape Remote Sensing Atmospheric Profile and Evapotranspiration eXperiment (GRAPEX) project. Daily, seasonal, and inter-seasonal surface flux patterns and relationships across five vineyards over three distinct California wine production regions were investigated. Vineyard actual evapotranspiration showed significant differences at the sub-daily and daily scale when comparisons across wine production regions and varieties were performed. Water use in vineyards in the Central Valley was about 70% greater in comparison to the vineyards at the North Coast area due to canopy size, atmospheric demand, and irrigation inputs. Inter-annual variability of surface fluxes was also significant, even though, overall weather conditions (i.e., air temperature, vapor pressure deficit, wind speed, and solar radiation) were not significantly different. Thus, not only irrigation but also other management practices played a key role in seasonal water use, and given these differences, we conclude that further advancing ground-based techniques to quantify crop water use at an operational scale will be key to facing California’s agriculture present and future water challenges.

**Supplementary Information:**

The online version contains supplementary material available at 10.1007/s00271-022-00784-0.

## Introduction

Irrigation plays a key role in land surface fluxes and their interactions with atmospheric processes. Globally, it is estimated that the increase in water vapor fluxes from land due to irrigation is on the same order of magnitude as the decrease induced by deforestation (Gordon et al. 2005). Irrigated systems have expanded in recent years to increase water use efficiency and crop productivity, yet expected water savings have led to greater water demands due to agricultural expansion (Scott et al. 2014; Perry et al. 2017). Nearly 10% of California’s land surface area is irrigated farmland, where high-revenue perennial crops (e.g., nuts, grapes, and other fruit) account for about 40% of that land (Mount and Hanak 2016). Trends in the expansion of perennial crops as a replacement of annual crops are expected to result in a positive net surplus of water but will require policies and technologies to assure these benefits (Wilson et al. 2016).

California’s irrigated agriculture, and other semi-arid regions around the world, face growing threats by climate change, where an increase in precipitation variability is expected to result in more frequent, prolonged and extreme droughts (Persad et al. 2020). Regional climate models also project a challenging future related to natural water storage; by the end of this century water from snowmelt will occur 4 weeks earlier each year and also an expected 79% reduction in peak water volume would impact about 40% of California’s surface water storage (Rhoades et al. 2018). These water deficits are already being compensated for by increased reliance on groundwater to make up for surface water constraints, which is unsustainable at current rates of extraction (Stokstad 2020). Continually improving our understanding of water use by high-value woody perennial crops will enable growers to improve the precision irrigation management needed to achieve sustainability goals. In California, woody-perennial crop water use needed for irrigation management is commonly based on some combination of direct soil moisture measurements and indirect crop evapotranspiration estimates.

Actual ET (ET_a_) is the effective water flux from the surface to the atmosphere due to soil evaporation and plant transpiration. Thus, the evapotranspiration flux derived through micrometeorological methods such as eddy covariance and surface renewal can be regarded as estimates of ET_a_. Three related concepts are potential evapotranspiration (ET_p_), reference crop evapotranspiration (ET_o_), and crop evapotranspiration (ETc). ET_p_ represents an atmospheric water demand, thus the amount of water that can be transferred as water vapor to the air from the surface (i.e., land or water) (Thornthwaite 1948; Xiang et al. 2020). ET_o_ is commonly defined as the rate of evapotranspiration from a hypothetical crop with an assumed crop height (12 cm) and a fixed surface resistance (70 s/m) and albedo (0.23), which would closely resemble evapotranspiration from an extensive surface of green grass cover of uniform height, actively growing, completely shading the ground and not short of water(Allen et al. 1998). ETc is usually regarded as the evapotranspiration from a given crop depending on plant growth and other surface characteristics, and thus is a function of a crop coefficient representing such crop/surface characteristics and ET_o_ accounting for local weather conditions (Allen et al. 1998). ET_o_ is commonly obtained from a nearby weather station and multiplied by a crop coefficient to obtain ETc. The use of any of these approaches can lead to crop water use estimates with a significant amount of uncertainty. Such uncertainty is usually related to measurement quality and quantity, modeling assumptions, and logistical challenges of implementing these techniques in an operational context. While not absent of limitations and uncertainties, evapotranspiration fluxes derived through the eddy covariance technique can be considered as a direct measurement of crop water use. Moreover, eddy covariance flux measurements provide estimates at a temporal frequency that allows for the examination of sub-daily scale processes and relationships between different surface fluxes (Wilson et al. 2001).

The Grape Remote Sensing Atmospheric Profile and Evapotranspiration eXperiment (GRAPEX) project has collected micrometeorological and biophysical data in vineyards across two distinct California viticultural regions, North Coast and Central Valley, across several growing seasons. These regions not only exhibit different climatic conditions (Fig. S1), but are also characterized by different grapevine varieties, canopy structure, management practices, and production goals that represent a wide range of conditions encountered by California wine grape growers. In this study, we capitalize the rich GRAPEX eddy covariance dataset to better understand wine grape water use across this range of conditions.

GRAPEX aims to develop tools needed to remotely monitor ET_a_ and inform optimal irrigation management for a given vineyard based on detailed information regarding ET_a_ at high spatial and temporal resolutions (Kustas et al. 2018). This study contributes to the overarching objectives of GRAPEX by exploring eddy covariance surface flux variability for five different vineyards and highlights the value of near-real-time actual evapotranspiration as part of new irrigation management tools. Daily, seasonal, and inter-seasonal surface flux patterns and relationships are investigated. ET patterns and variability are analyzed along with factors closely tied to vineyard management, namely vegetation density as expressed by leaf area index (LAI) and water availability. LAI is related to vineyards phenology and influenced by vine training and pruning practices, as well as early-season irrigation, while water availability throughout the growing season is closely driven by irrigation practices.

## Methods

### Site description

**Fig. 1:**
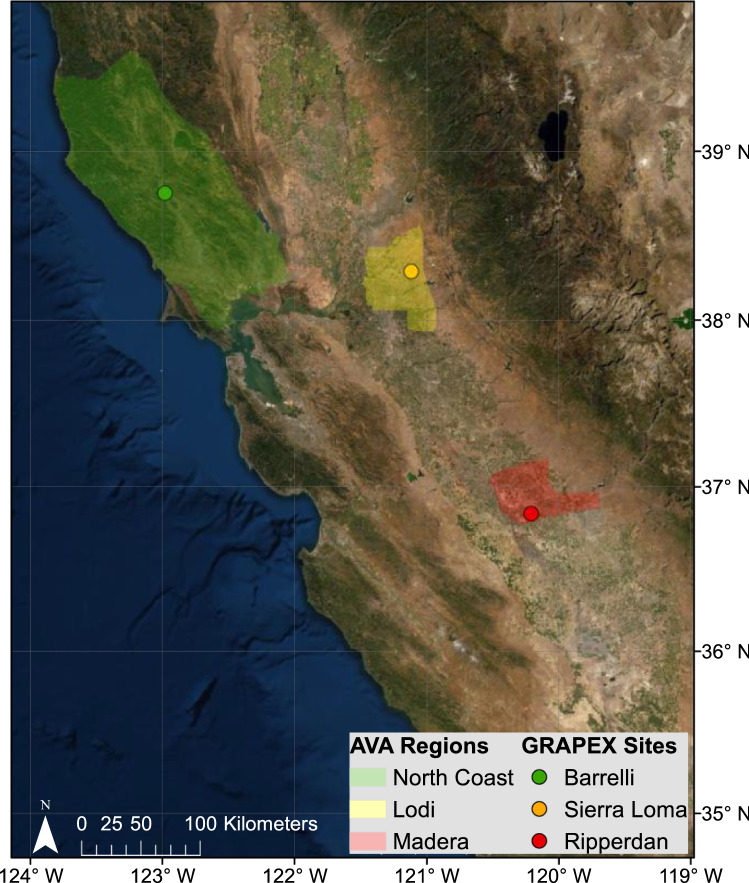
GRAPEX study sites and corresponding American Viticultural Area

Micrometeorological flux measurements were collected over five vineyards over the North Coast and Central Valley of California. According to the American Viticultural Areas boundaries, the three study sites where flux measurements were collected are located over the North Coast, Lodi, and Madera regions (Fig. 1). In the North Coast region, there were two flux towers deployed at the North Coast (BAR) study site: one in a Cabernet Sauvignon and another in a Petite Sirah vineyard. In the Lodi region, fluxes from a tower at the SLM study site in a Pinot Noir vineyard are presented for years 2018 and 2019, while in 2020 the block was converted to Cabernet Sauvignon by cutting the vines at the rootstock and re-grafting the former variety. In the Madera region, there were flux towers deployed at the RIP study site over vineyards with varieties Merlot and Chardonnay. Additional details regarding location, wine grape variety, soil texture, canopy type, etc. for each experimental vineyard are presented in Table 1.

**Table 1:** Study sites and vineyards characteristics

Study site	North Coast		Lodi	Madera	
Vineyard block identification	BAR_A07	BAR_A12	SLM_001	RIP_760	RIP_720 (Block #4)
Soil type	Gravelly loam	Gravelly loam	Loam/clay loam	Sandy loam	Loam/sandy loam
Vineyard characteristics					
Vine variety	Petite Sirah	Cabernet Sauvignon	Pinot Noir/Grafted to Cabernet Sauvignon	Chardonnay	Merlot
Year planted	2013	2010	2009/2020	2010	2010
Row orientation	Northwest–Southeast	Northeast–Southwest	East–West	East–West	East–West
Trellising method	Stack-T (Split canopy)	Elk-Horn (Split canopy)	Split canopy (quadrilateral)	Double Vertical	Bilateral cordon (split canopy)
Row width (m)	3.35	3.35	3.35	2.74	3.35
Planting interval (m)	1.83	1.83	1.52	1.83	1.52
Vine canopy height (m) (April–September)	1.5–2.2	1.5–2.3	2.0–2.75	1.5–2.5	1.5–2.2
Cover crop type	Annual mixed grass	Annual mixed grass	Annual mixed grass	Perennial grasses	Perennial grasses
Cover crop width (m)	2.75	2.75	2.00	1.20	1.85
Cover crop management	Periodic mowing (~ 3 times season)/cultivation alternate	Mowed once or twice in April/May	Mowed once or twice in April/May	Mowed once or twice in April/May	Mowed once or twice in April/May
Irrigation system	Drip irrigation (2 × 2 L/h flow rate emitters)	Drip irrigation (2 × 2 L/h flow rate emitters)	Dripline at the center of the vines (0.25 m agl). Dripper at 0.35 m distance from each side of the vine	In line dripper (3 × 2 L/h flow rate emitters per vine)	Variable Drip Irrigation (VRDI) (3 L/h flow rate emitters)
Flux tower installation date	4/4/19	5/12/17	4/2/13	5/9/17	4/9/18

### Instrumentation

Flux towers at each site were equipped with sensors to measure the main components of a surface energy balance (e.g., Net radiation (*R*_*n*_), Sensible heat flux (*H*), Latent heat flux *(λE*), and soil heat flux (*G*)). Each tower had a very similar array of sensors, yet some differences were unavoidable due to the availability of sensors at the time of deployment and changes in technology throughout the deployment at the different study sites. A detailed list of the sensors deployed at each flux tower is presented in Table 2. The soil heat flux was calculated as the average of five heat flux plates deployed along a diagonal transect across the inter-row space at a depth of 8 cm. At each soil heat flux plate location, soil thermocouples at depths of 2 cm and 6 cm, and a soil moisture sensor at a depth of 5 cm were installed.

**Table 2:** Flux towers instrumentation

Sensor type	Vineyard block identification				
	BAR_007	BAR_012	SLM_001	RIP_760	RIP_720
Net radiometer	SN-500, Apogee Instruments, *z* = 4.5 m	NR01-L, Hukseflux, *z* = 6.5 m	CNR1, Kipp & Zonen, *z* = 5.0 m	NR01-L, Hukseflux, *z* = 6.5 m	NR01-L, Hukseflux, *z* = 5.0 m
3D Sonic anemometer	CSAT3, Campbell Scientific, *z* = 4.5 m	CSAT3, Campbell Scientific, *z* = 5.0 m	CSAT3, Campbell Scientific, *z* = 5.0 m	CSAT3, Campbell Scientific, *z* = 5.0 m	Integrated sonic anemometer and gas analyzer, IRGASON, Campbell Scientific, *z* = 4.5
Infrared gas analyzer	EC150, Campbell Scientific, *z* = 4.5 m	EC150, Campbell Scientific, *z* = 5.0 m	EC150, Campbell Scientific, *z* = 5.0 m	EC150, Campbell Scientific, *z* = 5.0 m	
Air temperature and humidity probe	HMP45C, Vaisala, *z* = 4.5	EE08 (E + E Elektronik) probe in aspirated shield TS-100 (Apogee Instruments), *z* = 5.0	EE08 (E + E Elektronik) probe in aspirated shield TS-100 (Apogee Instruments), *z* = 5.0	EE08 (E + E Elektronik) probe in aspirated shield TS-100 (Apogee Instruments), *z* = 5.0	EE08 (E + E Elektronik) probe in aspirated shield TS-100 (Apogee Instruments), *z* = 4.5
Soil heat flux sensor	HFT-3, Radiation Energy Balance Systems	HFT-3, Radiation Energy Balance Systems	HFT-3, Radiation Energy Balance Systems	HFT-3, Radiation Energy Balance Systems	HFT-3, Radiation Energy Balance Systems
Soil thermocouple	Type E soil thermocouples	Type E soil thermocouples	Type E soil thermocouples	Type E soil thermocouples	Type E soil thermocouples
Soil moisture probe	HydraProbes (Stevens Water Monitoring System	HydraProbes (Stevens Water Monitoring System	HydraProbes (Stevens Water Monitoring System	HydraProbes (Stevens Water Monitoring System	HydraProbes (Stevens Water Monitoring System
Rain gauge	TE525, Texas Electronics		TE525, Texas Electronics	TE525, Texas Electronics	

The use of trade, firm, or corporation names in this article is for the information and convenience of the reader. Such use does not constitute official endorsement or approval by the US Department of Agriculture or the Agricultural Research Service of any product or service to the exclusion of others that may be suitable

### Flux data processing

*H* and *λE* were computed as functions of 30-min average covariance of the corresponding variables sampled at 20 Hz. Anomalous records in the high-frequency time-series for each computed variable were removed following the Median Absolute Deviation method implemented by Mauder et al. (2013). Wind velocity components were rotated into the mean streamwise flow following the 2-D coordinate rotation method described by Tanner and Thurtell (1969), Kaimal and Finnigan (1994), Foken and Napo (2008). When necessary, wind velocity and the scalar quantities were adjusted in time to account for sensor displacement, and frequency response attenuation corrections were performed (Massman 2000). Sonic temperatures were corrected based on Schotanus et al. (1983), and the resulting fluxes were adjusted by the Webb, Pearman, and Leuning (WPL) density corrections (Webb et al. 1980). The five soil heat flux (*G*) measurements collected over the vineyard inter-row were corrected to represent a surface approximation by accounting for the heat storage in the overlying soil layer. Once *G* observations were corrected to the surface level, the individual half-hourly measurements of the soil heat flux were averaged to produce a representative *G* flux for each vineyard (Agam et al. 2019).

### Leaf area index

The leaf area index (LAI) of vines and interrow cover crop, when it is green and active, was estimated on a 30-m spatial scale for the GRAPEX project using satellite imagery following the method developed by Gao et al. (2012). This method is based on using a regression tree on homogenous MODIS LAI retrievals to obtain LAI estimates at 30-m resolution from the reflectance bands measured by Landsat. This approach has been previously evaluated within the GRAPEX project, and it has proven to provide reliable estimates compared to ground-based LAI measurements derived from LiCor LAI-2000 and LAI-2200 measurements (White et al. 2019).

### Statistical analyses

Statistical analyses were performed in python built upon the SciPy library. Linear least-squares regressions were performed to compared meteorological variables [i.e., air temperature, vapor pressure deficit (VPD)] and daily surface fluxes (*R*_*n*_*, H,* λE, and *G*) between the five flux towers aforementioned. Furthermore, differences in the measured fluxes across the vineyards were characterized by mean difference (MD), mean absolute differences (MAD), and root mean square difference (RMSD) statistics (Alfieri et al. 2019). Statistical significances were tested using the Mann–Whitney *U* test since this nonparametric test does not assume a normal distribution (Wilks 2011).

Linear least-squares regressions were performed to compare daily ET_a_ and ET_o_ fluxes. ET_a_ fluxes were computed as daily sums of sub-daily eddy covariance fluxes (30 min.) corrected by adding the corresponding Bowen fraction of residual energy due to lack of energy balance closure. ET_o_ was retrieved from the California Irrigation Management Information System (CIMIS). CIMIS stations were chosen based on proximity to the GRAPEX study sites and common use by growers in these regions.

## Results and discussion

**Fig. 2:**
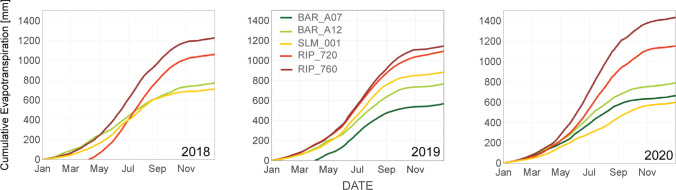
Cumulative evapotranspiration from eddy covariance flux measurements from 5 different GRAPEX study sites. RIP_720 and BAR_A07 are integrated in the figure once those flux tower were installed, so the cumulative values for those sites do not represent a full year until 2019 and 2020, respectively

**Fig. 3:**
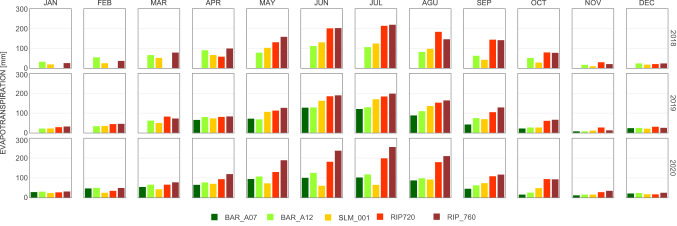
Monthly total ET from eddy covariance flux measurements from 5 different GRAPEX study sites

ET_a_ showed distinct daily, seasonal, and inter-annual patterns across vineyards located in different viticultural areas. Cumulative annual evapotranspiration from vineyards in California’s Central Valley (Madera AVA Region) was about 70% larger than the observations for the North Coast vineyards (Fig. 2). Overall, total seasonal evapotranspiration had very similar magnitudes within vineyards of different varieties and trellising systems located in a proximal location, yet total annual ET_a_ at the North Coast vineyards exhibit differences greater than 30%. High ET_a_ fluxes over California’s Central Valley were expected due to larger atmospheric evaporative demands (Fig. S2), yet the magnitude of differences found can only be explained by also accounting for irrigation and other agricultural management practices (e.g., cover crop, hedging, and yield targets). In the Madera region, vineyards' water use based on growing season accumulated ET_a_ by the end of May is equivalent to the magnitude of precipitation from the previous winter season. Consequently, atmospheric water demands during the summer months have to be satisfied through irrigation. In the summer (i.e., June, July, and August), ET_a_ was significantly larger in the Madera vineyards, yet the magnitude of these differences changed throughout the three years analyzed in this study (2018–2020) (Fig. 3). The impact of viticultural practices on vines' water use can only be measured directly and timely for irrigation management using ground-based sensors. Satellite remote sensing can provide daily ET_a_ estimates at 30 m spatial resolution at most every eight days using Landsat in the best-case scenario, although fusion techniques using multiple satellite sources can extrapolate to daily estimates, but with greater uncertainty (Knipper et al. 2019). However, such latency and uncertainty in modeled remote sensing information could be overcome by combining economical ground-based approaches such as surface-renewal (Spano et al. 2000; Parry et al. 2019) along with satellite-based ET_a_*.*

Differences across study sites are expected based on the variety of soils, plant density, trellising systems, plant varieties, and geographic locations (Table 1). Such variability is observed across the analyzed results, yet certain characteristics patterns emerge as well. ET_a_ fluxes while linked to meteorological conditions, they are heavily influenced by viticultural management and production goals in terms of yield and fruit quality. Given the complex interactions between soil characteristics, weather conditions, vine varieties, phenology, and management, this study highlights the importance of advancing tools to inform irrigation management based on ET_a_. We argue that while ET_c_, ET_p_, and ET_o_ are also important, these variables do not provide information regarding local conditions and vineyard responses as feedback to management practices. Throughout this study, we have also found a nuanced relationship between specific viticulture management practices and vines’ water use. For instance, the timing, frequency, and magnitude of hedging and cover crop mowing led to different ET_a_ responses throughout the assessed years and study sites.

### Meteorological conditions comparison

**Fig. 4:**
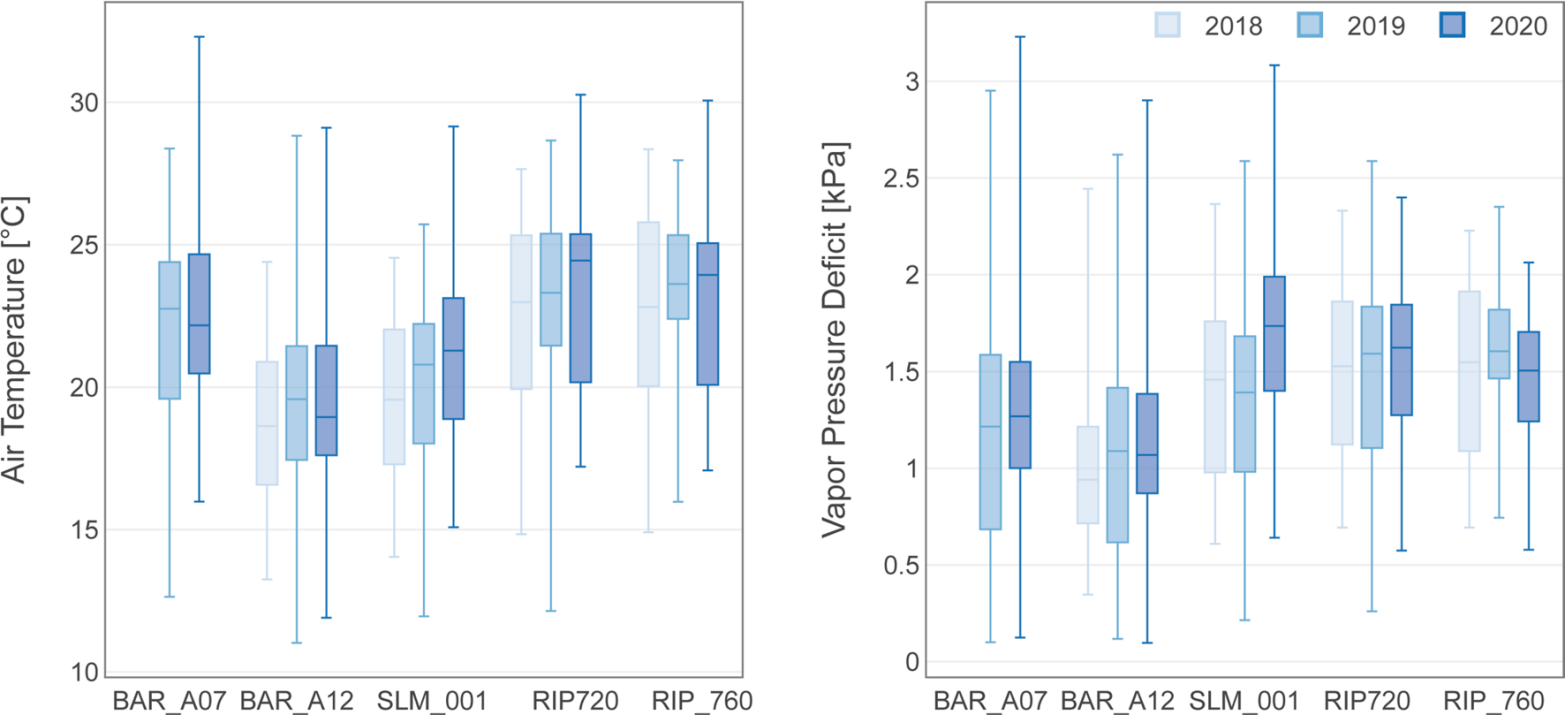
Box-and-whisker plots (i.e., minimum, 25th percentile, median, 75th percentile, and maximum) for daily mean temperature and vapor pressure deficit for each study site and analyzed year

**Fig. 5:**
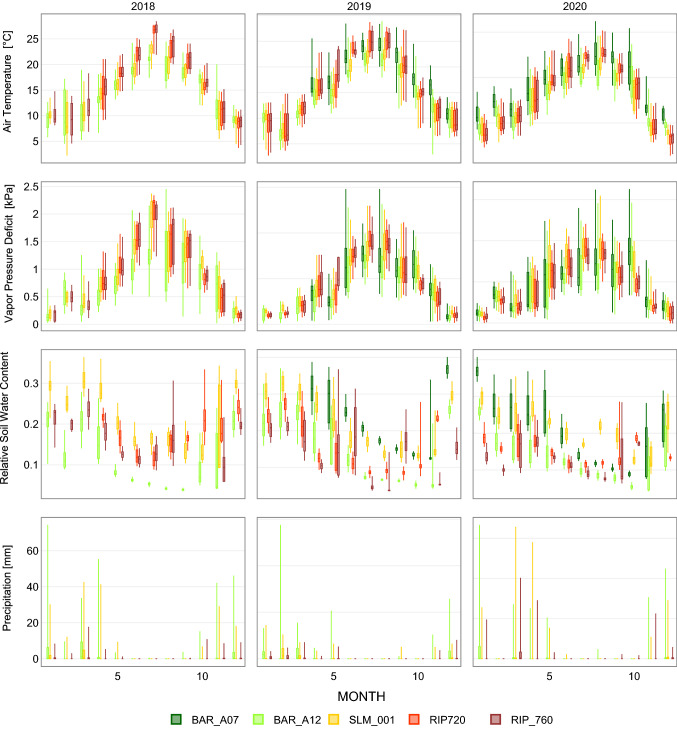
Monthly box-and-whisker plots (i.e., minimum, 25th percentile, median, 75th percentile, and maximum) for daily mean temperature, vapor pressure deficit, relative soil water content and precipitation

Meteorological conditions observed at the vineyards varied across different viticultural regions, and such differences are within the magnitude of the regional meteorological conditions observed in the respective viticultural areas (Fig. S1). At each site, statistical analysis of mean daily temperatures and vapor pressure deficits do not suggest significant differences (*p* > 0.1) between years at each vineyard, which suggest that interannual variability in overall meteorological conditions did not significantly affect atmospheric water demands throughout the analyzed growing seasons (Fig. 4). As an exception, significant differences were observed in VPD and to a lesser degree in air temperature for the SLM site for the 2020 growing season as compared to the previous years. This difference is probably related to the re-grafting of the vines that occurred in the winter of 2020, which caused a significant reduction in *λE* and resulted in higher *H*, thus contributing to higher local VPD and air temperature. Differences in temperatures and atmospheric moisture measured at the North Coast vineyards (BAR_A12 and BAR_A07) were influenced in part by the absence of an aspirated shield in the BAR_A07 vineyard (Huwald et al. 2009). Observed air temperature and atmospheric moisture closely resemble historical climate conditions at each study region, yet more days with extremely high temperatures and low atmospheric humidity were recorded during the analyzed period. As expected, observed air temperature and atmospheric water vapor increased and decreased, respectively, from the northern to the southern vineyards (Fig. 5). When comparing summer months air temperature and VPD between the analyzed vineyards, we found larger differences than during the rest of the year, which we attribute to the influence of management, especially irrigation, on the local observed conditions.

During the growing season, weather patterns show consistency throughout the analyzed viticultural regions (Fig. 5). Linear regression analyses indicate significant linear relationships even between vineyards in the North Coast and Madera (Fig. S2 and S3), yet there is a difference in magnitude of temperature and VPD as mentioned above. Precipitation decreases towards the South, yet in March and April 2020, some large precipitation events took place over the Central Valley. However, based on relative soil water content observation at the SLM and RIP vineyards, these events do not seem to have affected long-term soil moisture conditions (Fig. 5).

Overall, greater near-surface relative soil water contents are observed in the BAR and SLM vineyards when compared to moisture conditions in the Madera fields (Fig. 5), yet during the growing season, one of the vineyards in BAR (BAR_A12) reaches the driest conditions by mid to late summer. In contrast, the SLM vineyard remains wetter than the other vineyards throughout the year. The observations also suggest a larger range in soil moisture during the winter months, when most of the soil water relies on precipitation events. However, in August of the analyzed years, large dispersion in soil moisture was consistently observed in RIP_760 as well. This condition is related to flood irrigation events during that time of the year, after harvest. This is followed by a large increase in soil moisture at RIP_720 in November which is typically flood irrigated for redistributing salts and preparing for replanting of the cover crop. Flood irrigation events represent a considerable amount of the total water supply annually, which we have quantified as about to represent near a third of the total annual crop water use in the Madera vineyards. The nature of flood irrigation imposes an additional level of uncertainty given the variability in infiltration capacity across the field. Unfortunately, the total amounts applied were not closely monitored by the growers. We speculate that vines might be able to tap into water stored deep in the soil when drip irrigation is not able to fully satisfy atmospheric demands early in the summer. While a dense array of soil moisture sensors up to 0.9 m deep is deployed in the Madera sites, the observations seem to indicate that water infiltrates deeper in the soil (data not shown) which compounds with the abovementioned uncertainties. Consequently, this highlights the need for monitoring vine physiological stress indicators early in the growing season as a key information piece for irrigation management.

### Comparison of surface fluxes

**Fig. 6:**
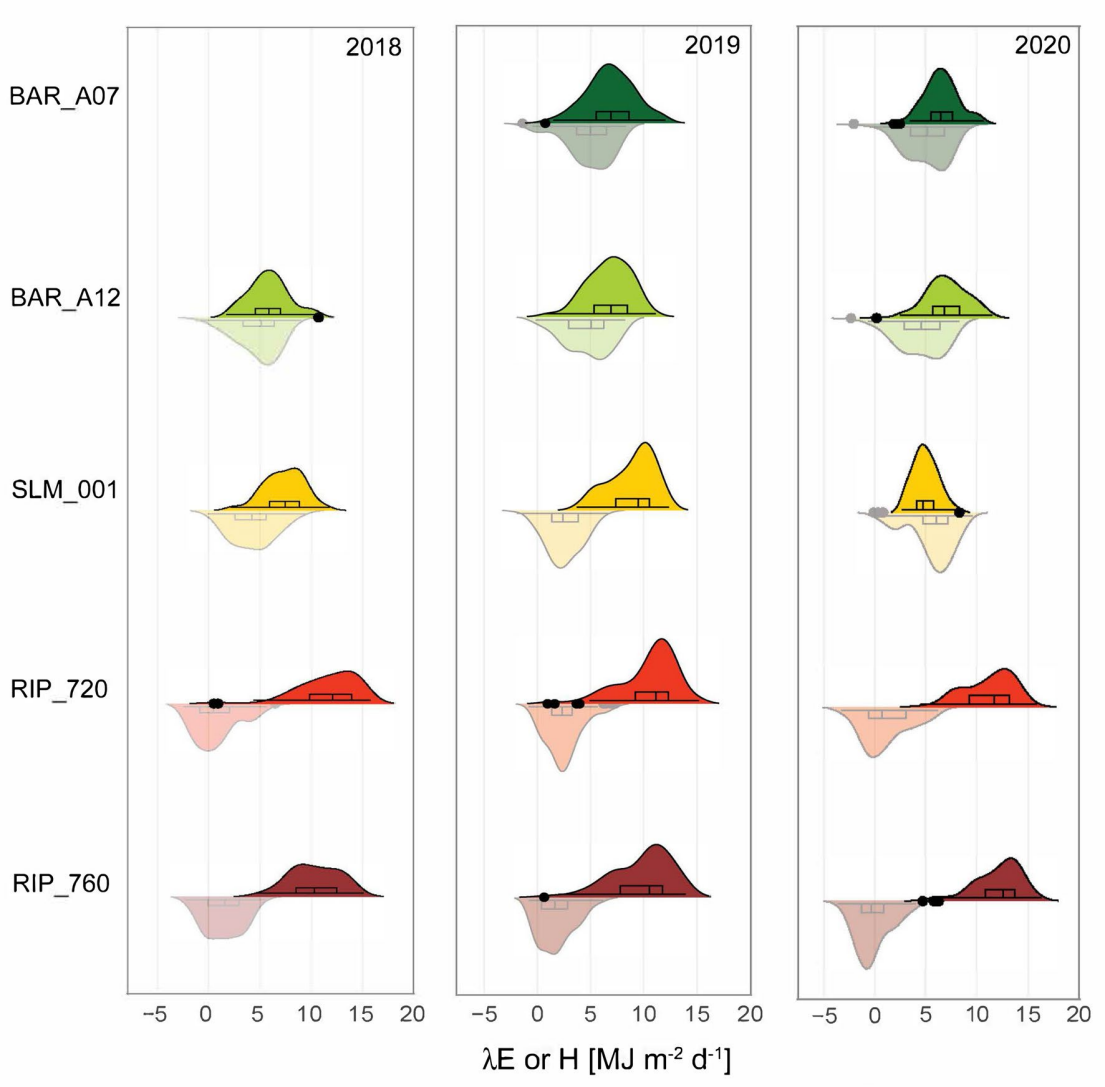
Violin plots illustrating daily λ*E* (solid color and top distribution) and *H* (clear color and bottom distribution) during the growing season (May–August). Box-and-whisker illustrate the minimum, 25th percentile, median, 75th percentile, and maximum flux per vineyard and year. Black dots represent outlier points located outside the whiskers of the box plot. Probability density plots depict fluxes distribution smoothed by a kernel density estimator

While median and mean daily *λE* and *H* during the growing season at each vineyard remain similar in magnitude throughout the study period, the distributions of these fluxes do not present similar patterns across years or within vineyards (Fig. 6). The Madera vineyards had a larger probability of low to negative *H*, which are largely compensated by high *λE* fluxes. Comparing the distribution of fluxes between BAR_A12 and RIP_760 provides a clear depiction of prevailing water stress conditions post-veraison in the North Coast vineyards versus near full satisfaction of atmospheric water demands in the Madera vineyards.

**Fig. 7:**
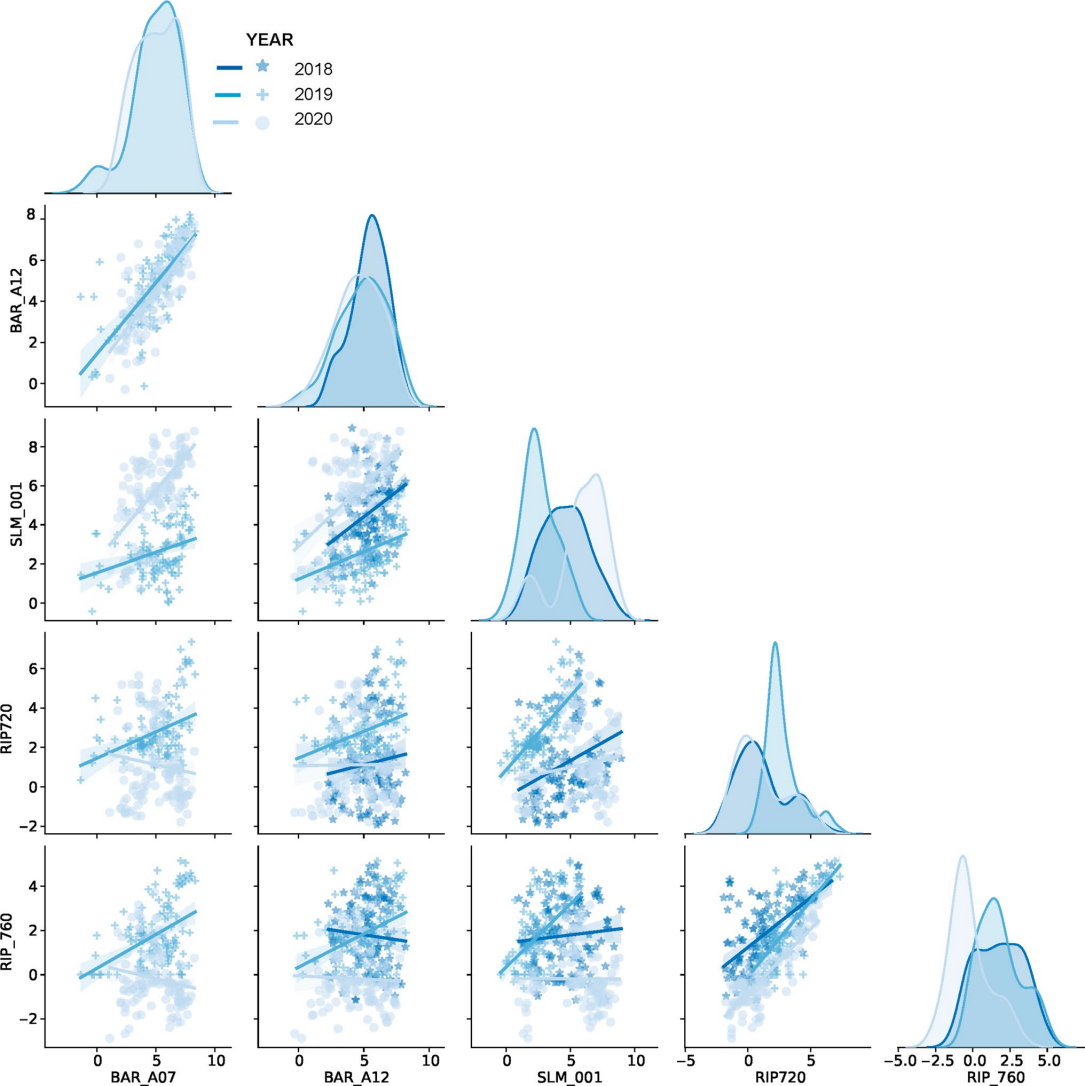
Comparison of daily net radiation (*R*_n_ in MJ m^−2^ day^−1^) flux between GRAPEX vineyards throughout the analyzed period (2018–2020). Solid lines represent derived linear least-squares regressions, and the respective parameters are listed in Table S1. Top diagonal charts illustrate the distribution of *R*_*n*_ at each site and analyzed year

Net radiation has a strong positive relationship between vineyards within a region, yet there were discrepancies throughout the growing season (Fig. 7). Observed differences were related to changes in the reflected shortwave radiation from the surface and longwave emitted terrestrial radiation. Those changes were usually related to management activities such as hedging of the vines or mowing of the cover crop. The relationship of net radiation between vineyards in different regions was not significant, and in some cases, negative to no linear trends were observed. These results highlight the importance of field-level four-component radiation measurements or advanced modeling approaches to accurately represent *R*_*n*_, especially when aiming to estimate other surface fluxes based on energy balance approaches (Parry et al. 2019). *G* estimates comparisons resulted in a slightly closer agreement across vineyards and within years (Fig. S4). Different cover crop practices in 2019 in RIP_720 (i.e., less frequent mowing leading to larger vegetative biomass throughout the season) led to a distinct soil heat flux pattern. Overall cooler soil surface temperatures led to a smaller amplitude of the variable throughout the day during the growing season, which illustrates the role of soil management in land surface energy fluxes partitioning. In addition to the results highlighted in this section, a detailed list of statistical parameters comparing surface fluxes and meteorological variables at a daily frequency across sites and analyzed years is presented in Table S1.

**Fig. 8:**
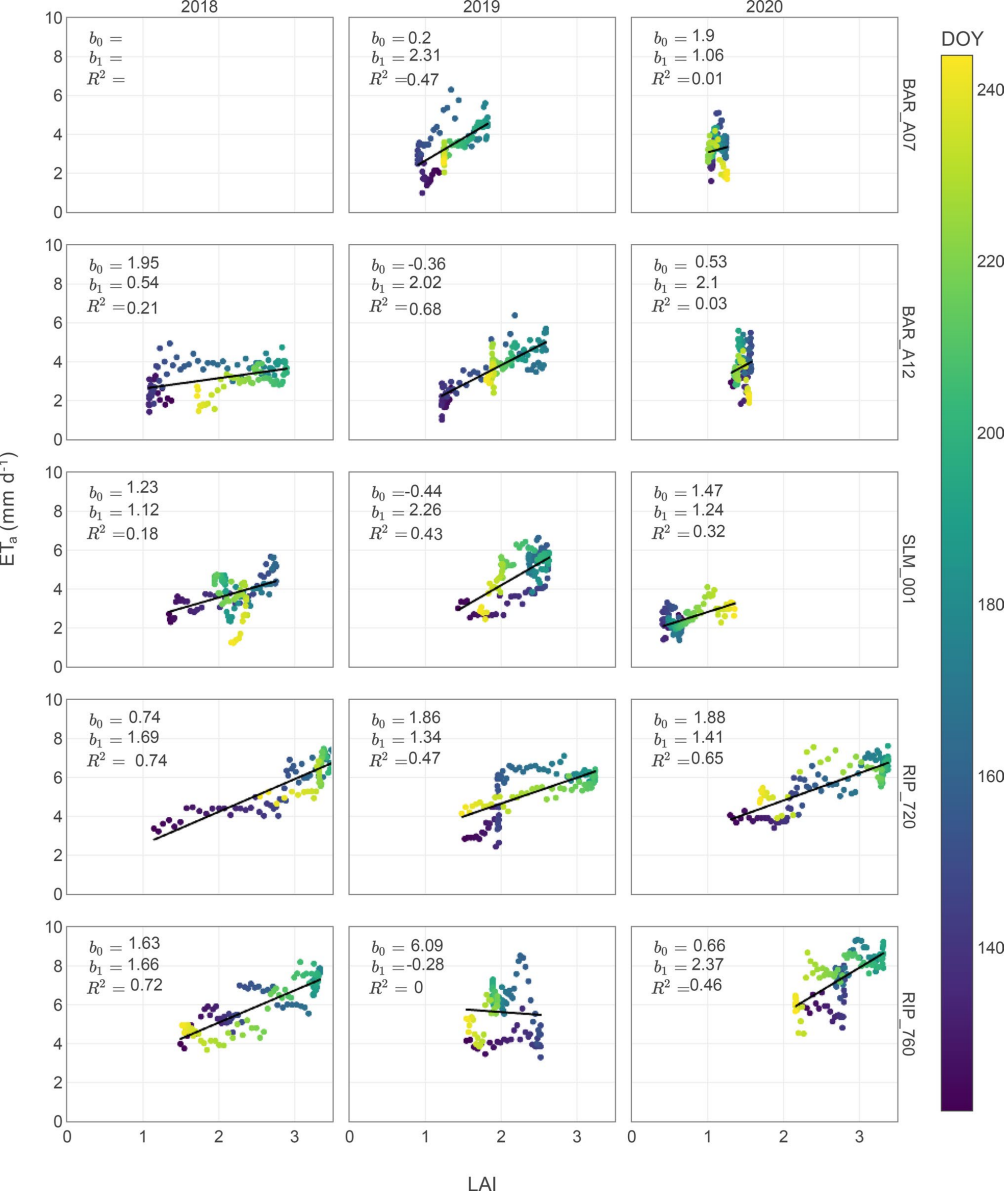
Leaf area index (LAI) and daily actual evapotranspiration (ET_a_) relationships. Colors represent the day of the year (DOY) and regression parameters (i.e., *b*_0_ = intercept, *b*_1_ = slope, and *R*^2^ = coefficient of determination) for linear least-squares regressions are presented for each site and analyzed year

LAI is commonly regarded as a key parameter related to plant canopy processes such as radiation interception, evapotranspiration, and carbon uptake (Welles and Norman 1991; White et al. 2019). The relationship between LAI and grapevines water use has been studied extensively (e.g., de Medeiros et al., 2001; Williams and Ayars, 2005), and it has been shown that trellising systems, vineyard density, pruning and hedging practices, and phenological stages, have an important effect in this relationship. Overall, we found that there was a positive relationship between LAI and ET_a_, yet the observed relationship shows dependency in phenological stages and particular characteristics at each vineyard. The vineyards analyzed in this study have different trellising systems, row orientations, vines densities, cover crop phenology and management (Table 1). The interactions of all these factors illustrate the complexity of the relationship between these variables (LAI and ET_a_). Slightly closer to linear relationships between LAI and ET_a_ were found mid to late growing season (~ DOY > 180), yet only in a few cases this relationship was statistically significant. Therefore, no clear patterns between LAI and ET_a_ were possible to distinguish from these analyses (Fig. 7). While in the same sites and years a stronger relationship between LAI and ET_a_ was observed (e.g., BAR_A12 in 2019, RIP_720 in 2018 and 2020, and RIP_760 in 2018), such behavior did not seem consistent throughout the study period. Large uncertainty is expected in vineyards LAI estimates based on satellite imagery, especially in vineyards with seasonal cover crops given the observed dramatic changes in relative foliage density and vertical distribution in time and space (Knipper et al. 2020). Thus, our results might suggest that extrapolating a given relationship between LAI and ET_a_ for calculations of a crop coefficient (*K*_c_) aiming to inform crop water demands might need to be carefully considered. In addition, in our analysis the presence of cover crops early in the season probably affects this relationship, indicating that considering this source of ET is also important (Fig. 8).

### Diurnal features of surface energy fluxes

**Fig. 9:**
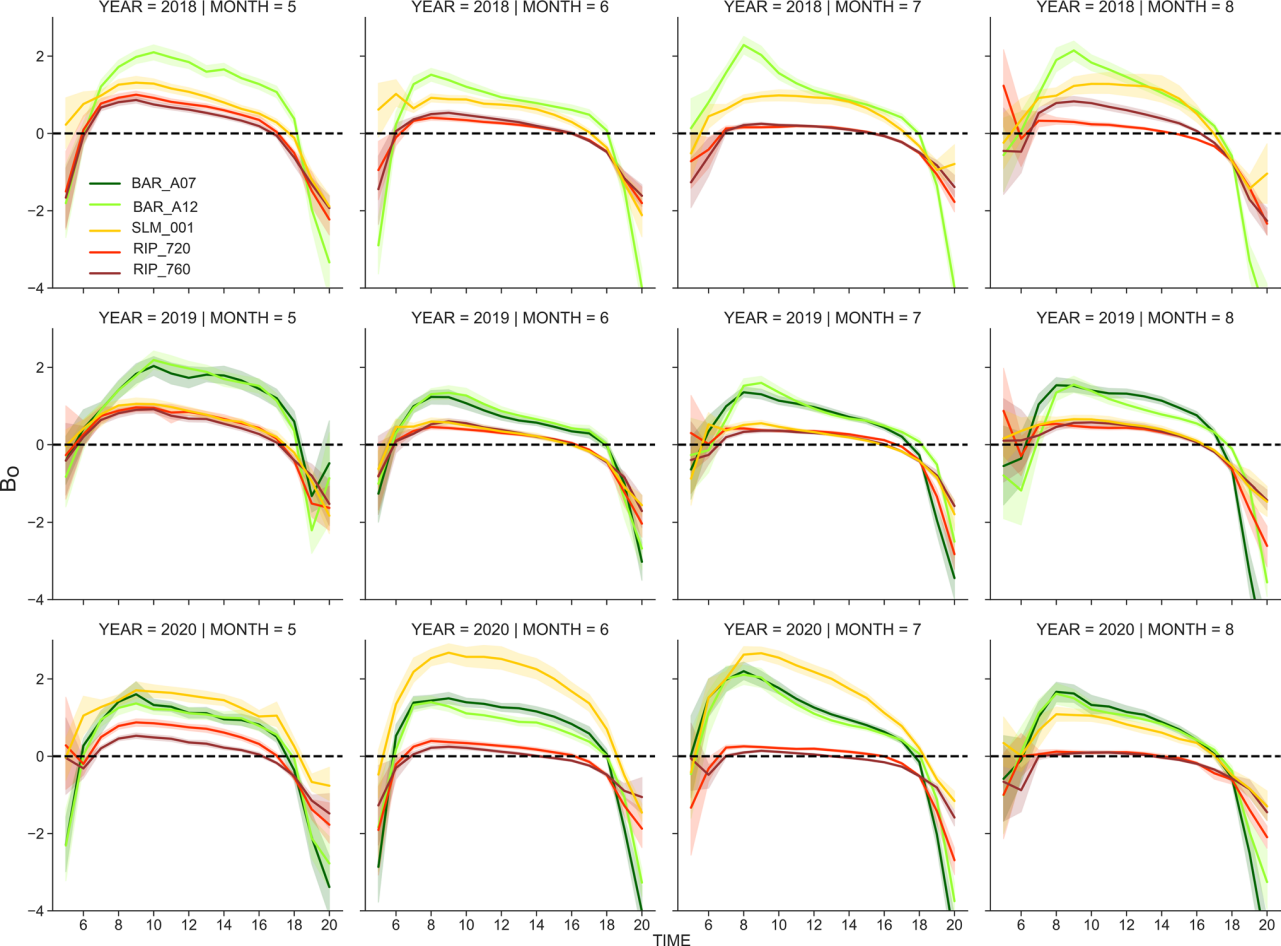
Mean diurnal Bowen ratios (*H*/λ*E*) per month during the growing season (May–August) for each site and analyzed year

Throughout the analyzed study period (2018–2020) and across sites, *λE* fluxes exhibited consistent diurnal features well coupled with solar radiation inputs (Fig. S5). However, diurnal *H (Fig. S6)* and Bowen ratios (*B*_*o*_) patterns (Fig. 9) suggest a potential ET enhancement due to advective conditions in June through August during the afternoon in the Madera vineyards. Under advective conditions, *H* is negative and behaves as an additional source of energy, which increases ET fluxes considerably when water is available. As part of the GRAPEX project, a new ongoing study is aiming to better understand and quantify the role of regional and local advection in ET_a_ and other surface fluxes in the Madera region.

Consistently low *B*_*o*_ (~ *B*_*o*_ < 1) throughout the growing season in the Madera vineyards suggest that ET fluxes remain near ET_p_ (Fig. 9). In contrast, larger estimated *B*_*o*_ in the North Coast vineyards might indicate water stress conditions, especially at midday during July and August, which is consistent with the regulated deficit stress irrigation management targeted for these vineyards. In the SLM vineyard, *B*_*o*_ features different behaviors throughout the study period; moderate water stress conditions in 2018, no stress in 2019, and considerably high *B*_*o*_ in 2020 are related to a small canopy due to the re-grafting that took place that year, which is consistent with low observed LAI values (Fig. S7).

### Contrasting production goals imprinted in vineyards surface energy fluxes

Regulated deficit irrigation (RDI) techniques are an essential component of viticultural management for wine grape production. Fine-tuned irrigation goals in viticulture do not only focus on timely satisfying plant water demands but also manipulating water stress to accomplish production goals in terms of yield and fruit quality. In grapevines, shoot growth is extremely responsive to water stress, therefore canopy growth can be controlled by deficit irrigation. Furthermore, fruit size at harvest can be controlled by water deficit during pre-veraison, usually recommended by prescribing some degree of water deficit for a short period soon after fruit set (Matthews et al. 1987; McCarthy et al. 2002). Conversely, early season water deficits are typically avoided due to the risk of poor fruit set (Keller et al. 2008), and irrigation is often ramped up at the end of the growing season to prevent root damage due to low soil moisture at dormancy in some regions (Keller et al. 2008). Based on our observations, the North Coast vineyards effectively use irrigation to regulate water supplies, yet in the vineyards over the Lodi and Madera regions irrigation closely satisfies atmospheric water demands. Different viticultural strategies across vineyards are also noticed by the trajectories observed in LAI (Fig. S7). Unstressed water conditions led to significantly larger canopies in the Central Valley vineyards, which play as feedback in terms of water demands. Thus, larger canopies have a greater transpiration potential, which is well satisfied based on our observations.

Different production goals in terms of yield and fruit quality underline the magnitude of targeted irrigation inputs across California’s viticultural regions. While irrigation management in viticulture aims to balance maximizing yield and achieving high-quality fruit, different regions are subject to different fruit quality expectations. Such expectations are related to reputation and the overall expected fruit quality potential recognized as terroir (Leeuwen and Seguin 2006). Growers in the North Coast focus on quality to satisfy the demand to produce high-value wines. In the Madera region, a focus on high yield is more prevalent since the area does not seem to have a special recognition for wine production. The Lodi area has some recognition as an emerging wine region, therefore production goals are not as biased towards yield or quality as in the former regions. Vine water use throughout the season is largely supported by irrigation. ET_a_ fluxes represent vine water use across different viticultural regions and illustrate the use of irrigation management as a tool to accomplish distinct production goals within a viticultural production program. For instance, growing season water use in the North Coast is about half in comparison to vineyards in the Madera region, and so it is the expected resulting yield.

### Ground-based actual ET is a key component to fine-tune vineyard irrigation management

Based on our results, for any given meteorological condition, we would expect a wide range of ET_a_ depending on the different aspects of viticultural management. Therefore, further developing tools that can accurately estimate plant water use and stress has been addressed as a key aspect of further advancing ET modeling methods and irrigation tools (Knipper et al. 2019). As vineyards’ water demands highly depend on production goals and viticultural management, it seems unlikely that any derivation of ETc or ET_o_ could provide enough information to effectively fine-tune irrigation while controlling stress levels.

**Fig. 10:**
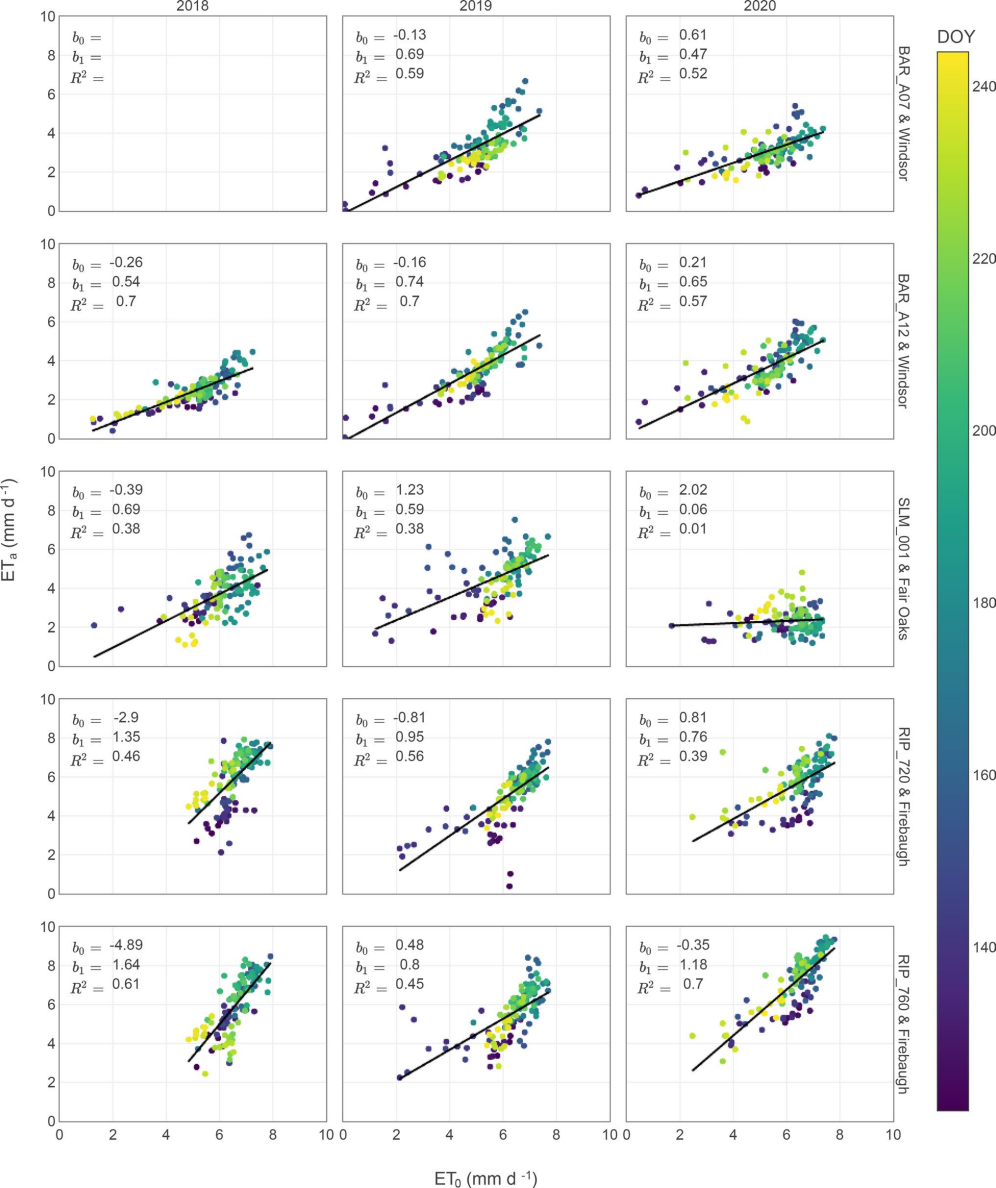
Daily reference and actual evapotranspiration comparison for GRAPEX vineyards. Reference evapotranspiration is based on data from the California Irrigation Management Information System (CIMIS) for stations near the GRAPEX study sites (Windsor—ID#103, Fair Oaks—ID#131, and Firebaugh—ID#7)

A comparison of daily ET_o_ and ET_a_ for vineyards in the three viticultural areas analyzed in this study shows that there is not a reliable relationship between these two variables across sites and years (Fig. 10). When assuming a linear response between ET_o_ and ET_a_ is not possible to distinguish a consistent pattern in the regression coefficients underlying these relationships. The intercept (*b*_*0*_) range is greater than 5 mm day^−1^ while the slopes (*b*_*1*_) could lead to ET_a_ estimates of near half to twice ET_o_. Independent of a given viticultural production goal, our results highlight the need for accurate estimates of ET_a_. Estimates of ET_a_ in combination with ET_o_ can provide a clear depiction of the amount of water demanded by a vineyard in comparison to a reference well-irrigated crop. Then, irrigation management could aim at a consistent ratio of these two variables that would satisfy plant water demands and regulate stress when needed.

For the studied sites within the GRAPEX project, water managers in the North Coast vineyards might have more water available than their counterparts in the Central Valley; they routinely require accurate *ET*_*a*_ information to regulate vine water stress to optimize the relationship between fruit quality and yield. On the other hand, when yield is a priority, such as in many vineyards across California’s Central Valley, water demands can be much higher. In these cases, optimizing irrigation to satisfy atmospheric demands throughout an entire growing season becomes more relevant, especially during drought years.

**Fig. 11:**
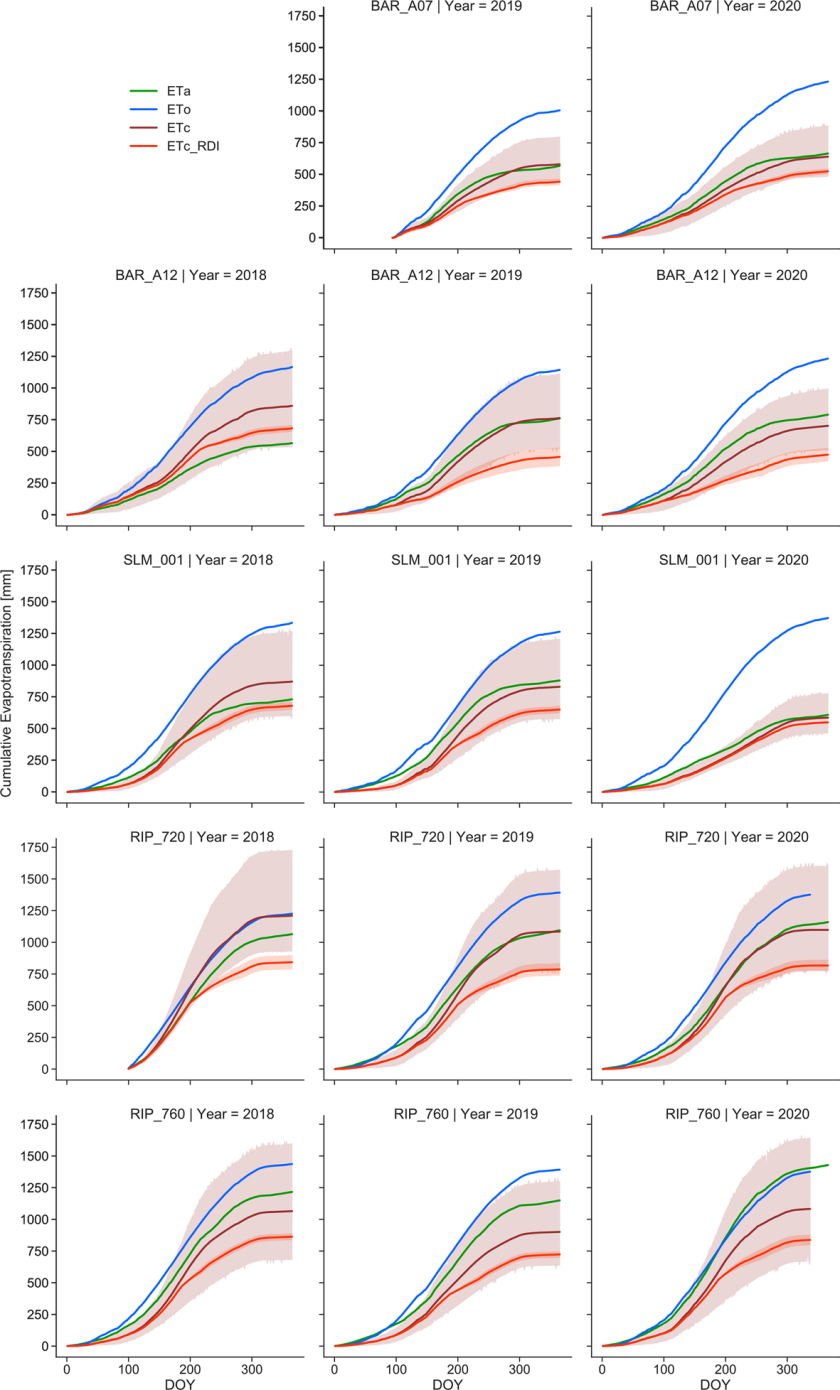
Cumulative ET_a_, ET_0_ (CIMIS), ETc, and ET_c_RDI_ comparison for GRAPEX vineyards throughout the analyzed period (2018–2020). ETc is depicted as the mean estimates (solid dark-red line) of four different approaches to derive vineyard crop coefficients (Williams and Ayars 2005; Netzer et al. 2009; Carrasco-Benavides et al. 2012; Munitz et al. 2019) and the dark-red shaded region represents the area between the 10th and 90th percentiles. RDI strategies were estimated as 50–75% of the mean ETc and are depicted as a red shaded region and the respective mean value in a solid red line

A comparison of daily ET_a_, ETc, ET_0_, and vine water demands following RDI strategies shows that irrigation management can lead to a wide range of vine water use (Fig. 11 and S8). Our results also show that in all study sites, ET_a_ tracks closely or is usually above ETc*,* indicating that limited stress conditions are prescribed across these vineyards. Consequently, important water-saving opportunities could be possible if RDI strategies were effectively implemented. Considering the time–frequency and latency resulting from satellite remote sensing ET_a_ estimates, ground-based sensors can offer a unique complement to timely inform irrigation decisions within an RDI program. Unfortunately, there is limited availability of commercial products able to measure ET_a_, and further advancing these technologies might be a critical component to develop as part of irrigation management toolkits. Further studies within the GRAPEX project will integrate the relationship between water use and yield as well as fruit and wine quality for these vineyards. Those studies will aim to advance the understanding of how much water is needed to achieve a given production goal, and also explore potential water-saving opportunities while not compromising yield and quality objectives.

## Conclusion

Land surface fluxes in vineyards are strongly shaped by irrigation, hedging, and cover crop management. While weather conditions drive *H* and *λE*, significant interannual variations seem more closely related to viticultural management. Based on the analyzed sites, which are part of the GRAPEX study, vineyards' water use in California’s Central Valley can be more than twice the ET_a_ observed in the North Coast vineyards. ET_a_ inter-annual variability within a given vineyard can also be significant, which resulted in differences greater than 300 mm in a year in the Madera area. The sensitivity of vineyards’ water use to management practices highlights the need for accurate ET_a_ estimates to achieve production goals and optimize water use. Unfortunately, other related variables such as ET_o_ or ETc seem not well suited to fine-tune irrigation given the challenges related to deriving specific crop coefficients and the lack of relationship found between ET_o_ and ET_a_. Therefore, further developing tools that can allow monitoring the ET_a_/ET_o_ ratio in relationship to plant physiological stress is a key aspect of the GRAPEX project goals. We found that effectively implement RDI could lead to important water-saving opportunities, which seems a clear path towards increasing the sustainability of irrigated viticulture. Ongoing studies are aiming to understand how growers could use this information (i.e., ET_a_ maps and leaf water potentials) when making irrigation decisions. We expect that developing low-cost, accurate, and timely estimates of ET_a_ would be fundamental to advance towards the sustainability of California’s agriculture in the context of climate change.

## Supplementary Information

Below is the link to the electronic supplementary material.

Supplementary file1 (DOCX 1927 kb)
