# Supplementary material for: Inter-annual variability of land surface fluxes across vineyards: the role of climate, phenology, and irrigation management
Source: Irrig Sci. 2022 Apr 15;40(4-5):463–80. doi: 10.1007/s00271-022-00784-0 (PMC9509312; doi:10.1007/s00271-022-00784-0)
Supplement: Supplementary file 1 — Supplementary file1 (DOCX 1927 kb) [file 271_2022_784_MOESM1_ESM.docx]

**Supplementary Material**


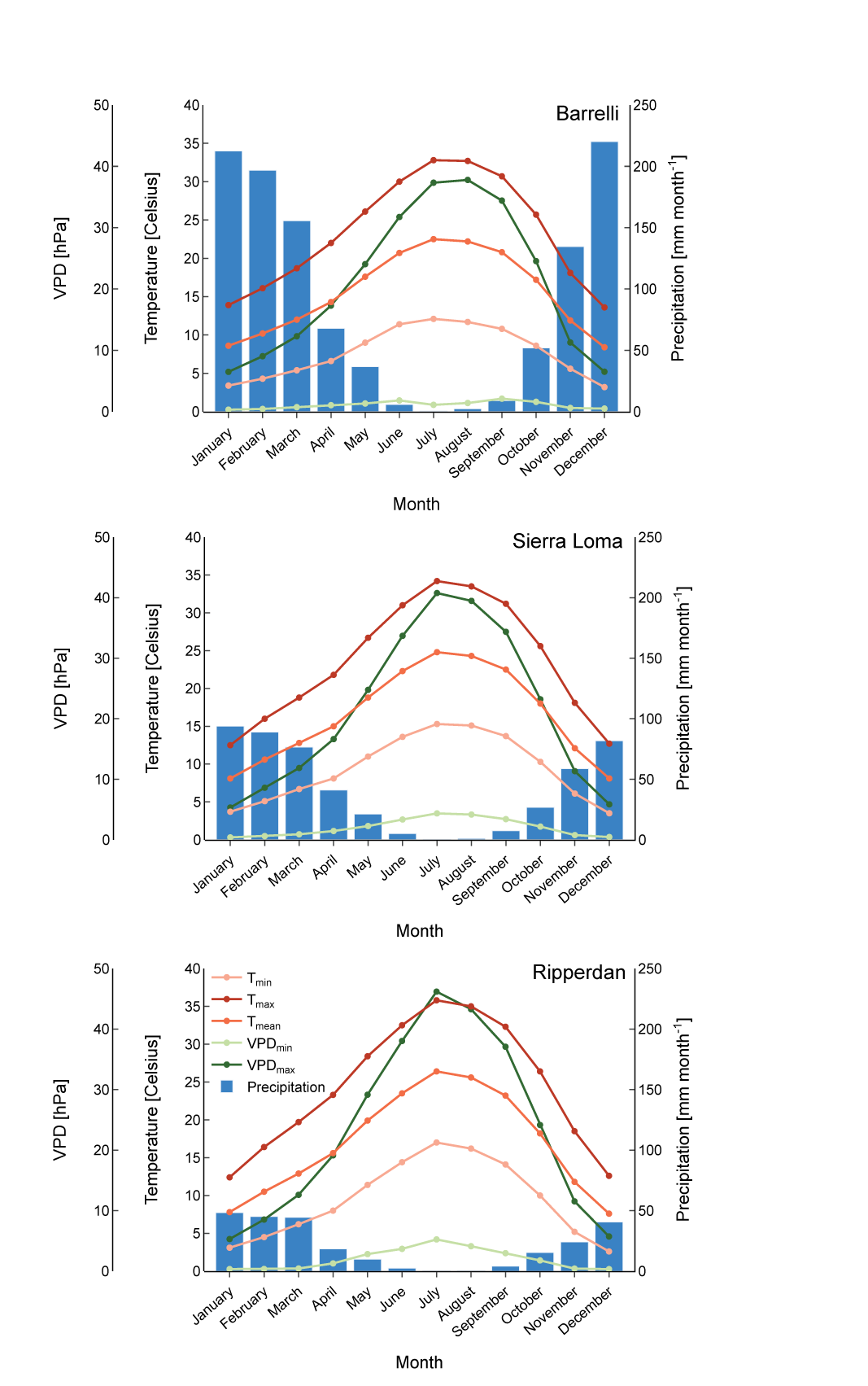


**Figure S1.** Climatological mean conditions (Temperature, Vapor Pressure Deficit (VPD) and Precipitation for the three GRAPEX study sites. PRISM^[[1]](#footnote-1)^ Monthly Mean Time Series Data, Period 1981 – 2010.

**
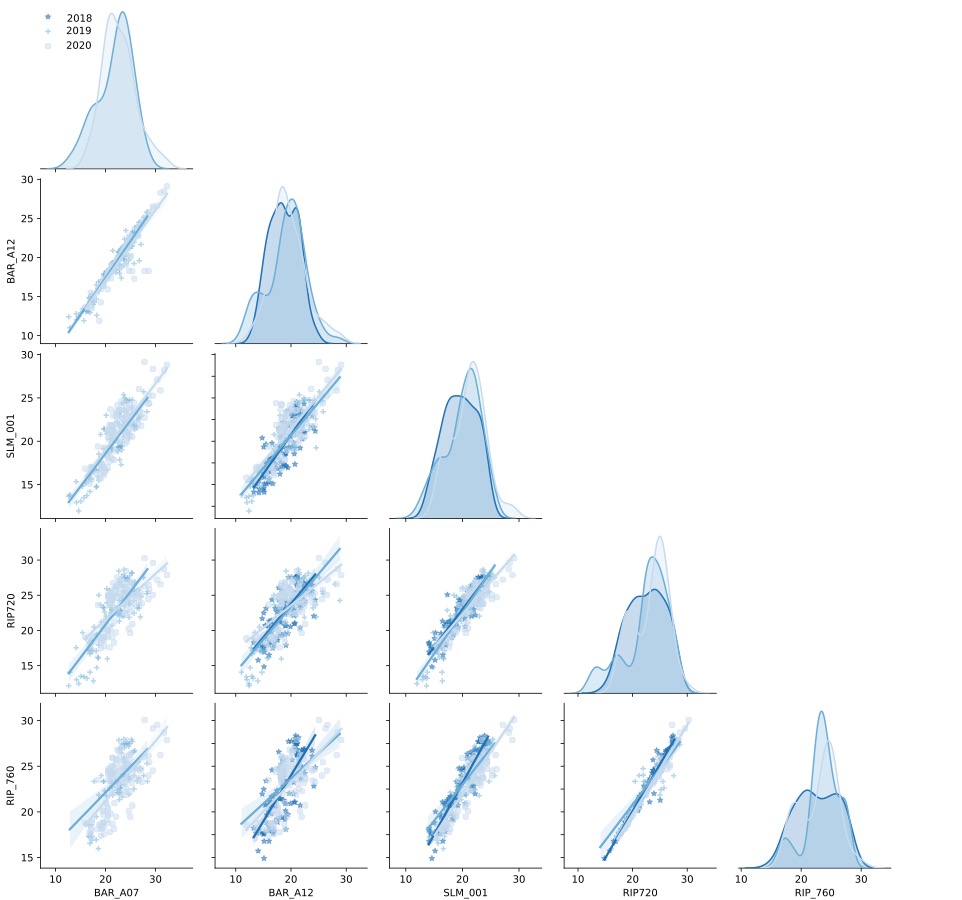
**

**Figure S2.** Comparison of daily mean air temperature (T_air_ in Celsius) between GRAPEX vineyards throughout the analyzed period (2018 -2020). Solid lines represent derived linear least-squares regressions, and the respective parameters are listed in Table S1. Top diagonal charts illustrate the distribution of T_air_ at each site and analyzed year.

**
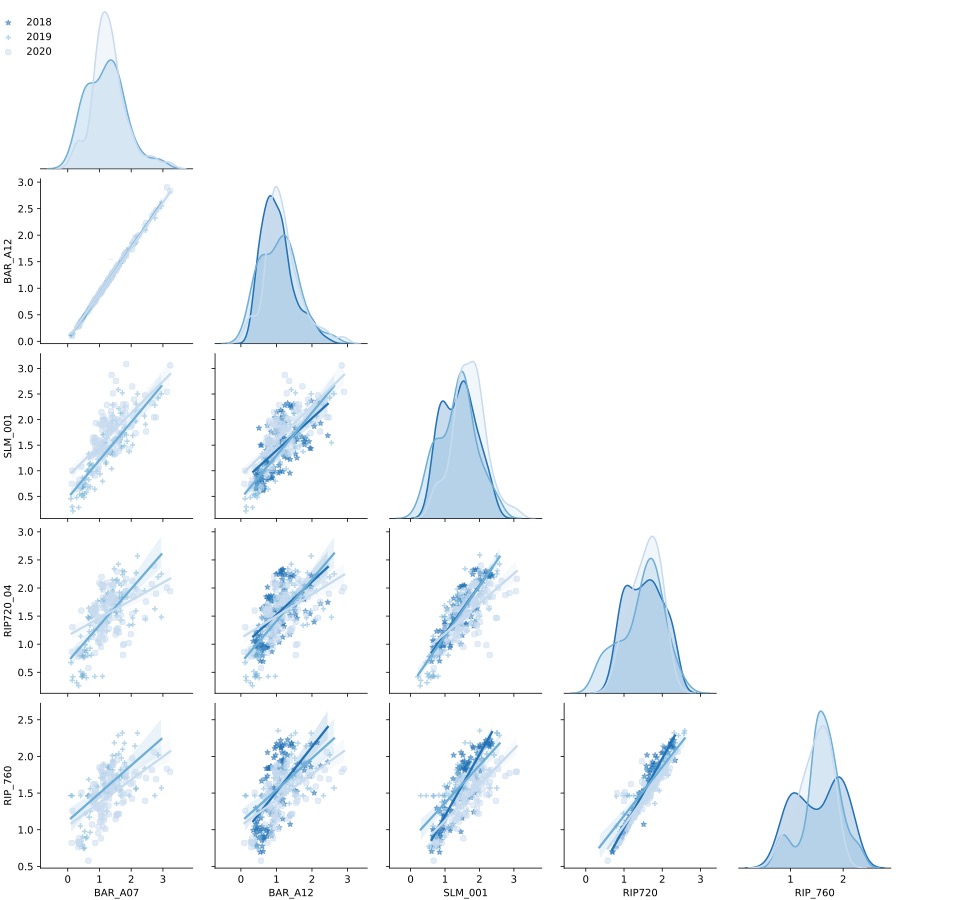
**

**Figure S3.** Comparison of daily air vapor pressure deficit (VPD in kPa) between GRAPEX vineyards throughout the analyzed period (2018 -2020). Solid lines represent derived linear least-squares regressions, and the respective parameters are listed in Table S1. Top diagonal charts illustrate the distribution of *VPD*  at each site and analyzed year.

**Figure S4.** Comparison of day-time soil heat flux sum (G in MJ m^-2^ d^-1^) between GRAPEX vineyards throughout the analyzed period (2018 -2020). Solid lines represent derived linear least-squares regressions, and the respective parameters are listed in Table S1. Top diagonal charts illustrate the distribution of *G* at each site and analyzed year.

**Figure S5.** Mean diurnal latent heat flux (λE) per month during the growing season (May – August) for each site and analyzed year.

**Figure S6.** Mean diurnal sensible heat flux (*H*) per month during the growing season (May – August) for each site and analyzed year.

**Figure S7.** Time series of Leaf Area Index (LAI) derived for vine and interrow cover crop for the 5 analyzed vineyards throughout the study period (2018 – 2020).

**Figure S8.** Daily ET_a_, ET_0_ (CIMIS), ET_c_, and ET_c_RDI_ comparison for GRAPEX vineyards throughout the analyzed period (2018 -2020). *ET_c_* is depicted as the mean estimates (solid dark-red line) of four different approaches to derive vineyard crop coefficients (Williams and Ayars 2005; Netzer et al. 2009; Carrasco-Benavides et al. 2012; Munitz et al. 2019) and the dark-red shaded region represents the area between the 10^th^ and 90^th^ percentiles. RDI strategies were estimated as 50% to 75% of the mean *ET_c_* and are depicted as a red shaded region and the respective mean value in a solid red line.

**Table S1. Statistical parameter of surface fluxes and meteorological variables comparisons.**

| Variable | Year | SITES | b_0_ | b_1_ | R^2^ | p | Standard Error | MD | MAD | RMSD | p  (U test) |
| --- | --- | --- | --- | --- | --- | --- | --- | --- | --- | --- | --- |
| ET | 2018 | BAR_A12 - RIP720 | 3.70 | 0.69 | 0.34 | 0.00 | 0.09 | -2.72 | 2.72 | 2.94 | 0.00 |
| ET | 2018 | BAR_A12 - RIP_760 | 3.34 | 0.79 | 0.39 | 0.00 | 0.09 | -2.67 | 2.67 | 2.90 | 0.00 |
| ET | 2018 | BAR_A12 - SLM_001 | 1.73 | 0.64 | 0.41 | 0.00 | 0.07 | -0.57 | 0.93 | 1.10 | 0.00 |
| ET | 2018 | RIP_720 - RIP_760 | 1.51 | 0.74 | 0.48 | 0.00 | 0.07 | 0.04 | 0.81 | 1.08 | 0.36 |
| ET | 2018 | SLM_001 - RIP_720 | 3.61 | 0.61 | 0.26 | 0.00 | 0.09 | -2.15 | 2.16 | 2.47 | 0.00 |
| ET | 2018 | SLM_001 - RIP_760 | 3.16 | 0.72 | 0.32 | 0.00 | 0.10 | -2.11 | 2.15 | 2.43 | 0.00 |
| ET | 2019 | BAR_A07 - BAR_A12 | 0.60 | 0.89 | 0.81 | 0.00 | 0.04 | -0.24 | 0.45 | 0.59 | 0.00 |
| ET | 2019 | BAR_A07 - RIP_720 | 2.39 | 0.84 | 0.55 | 0.00 | 0.07 | -1.86 | 1.92 | 2.08 | 0.00 |
| ET | 2019 | BAR_A07 - RIP_760 | 3.17 | 0.71 | 0.44 | 0.00 | 0.07 | -2.19 | 2.20 | 2.42 | 0.00 |
| ET | 2019 | BAR_A07 - SLM_001 | 2.04 | 0.79 | 0.58 | 0.00 | 0.06 | -1.33 | 1.37 | 1.57 | 0.00 |
| ET | 2019 | BAR_A12 - RIP_720 | 2.18 | 0.84 | 0.54 | 0.00 | 0.07 | -1.62 | 1.68 | 1.87 | 0.00 |
| ET | 2019 | BAR_A12 - RIP_760 | 2.92 | 0.73 | 0.46 | 0.00 | 0.07 | -1.95 | 1.97 | 2.19 | 0.00 |
| ET | 2019 | BAR_A12 - SLM_001 | 1.74 | 0.81 | 0.61 | 0.00 | 0.06 | -1.08 | 1.18 | 1.35 | 0.00 |
| ET | 2019 | RIP_720 - RIP_760 | 2.07 | 0.66 | 0.51 | 0.00 | 0.06 | -0.33 | 0.76 | 1.06 | 0.00 |
| ET | 2019 | RIP_720 - SLM_001 | 0.82 | 0.74 | 0.67 | 0.00 | 0.05 | 0.53 | 0.73 | 0.96 | 0.00 |
| ET | 2019 | RIP_760 - SLM_001 | 0.77 | 0.70 | 0.53 | 0.00 | 0.06 | 0.87 | 1.07 | 1.27 | 0.00 |
| ET | 2020 | BAR_A07 - BAR_A12 | -0.03 | 1.19 | 0.79 | 0.00 | 0.06 | -0.56 | 0.63 | 0.78 | 0.00 |
| ET | 2020 | BAR_A07 - RIP_720 | 3.58 | 0.68 | 0.21 | 0.00 | 0.12 | -2.59 | 2.59 | 2.83 | 0.00 |
| ET | 2020 | BAR_A07 - RIP_760 | 4.80 | 0.78 | 0.21 | 0.00 | 0.14 | -4.13 | 4.14 | 4.33 | 0.00 |
| ET | 2020 | BAR_A07 - SLM_001 | 2.00 | 0.12 | 0.03 | 0.07 | 0.07 | 0.69 | 1.01 | 1.19 | 0.00 |
| ET | 2020 | BAR_A12 - RIP_720 | 3.75 | 0.52 | 0.22 | 0.00 | 0.09 | -2.03 | 2.06 | 2.37 | 0.00 |
| ET | 2020 | BAR_A12 - RIP_760 | 4.89 | 0.64 | 0.24 | 0.00 | 0.10 | -3.57 | 3.59 | 3.81 | 0.00 |
| ET | 2020 | BAR_A12 - SLM_001 | 2.18 | 0.05 | 0.01 | 0.28 | 0.05 | 1.26 | 1.50 | 1.76 | 0.00 |
| ET | 2020 | RIP_720 - RIP_760 | 1.64 | 0.98 | 0.71 | 0.00 | 0.06 | -1.54 | 1.61 | 1.73 | 0.00 |
| ET | 2020 | RIP_720 - SLM_001 | 1.89 | 0.09 | 0.03 | 0.06 | 0.05 | 3.28 | 3.28 | 3.53 | 0.00 |
| ET | 2020 | RIP_760 - SLM_001 | 2.54 | -0.02 | 0.00 | 0.55 | 0.04 | 4.83 | 4.83 | 5.09 | 0.00 |
| H | 2018 | BAR_A12 - RIP720 | 0.28 | 0.17 | 0.01 | 0.22 | 0.14 | 4.22 | 4.26 | 4.82 | 0.00 |
| H | 2018 | BAR_A12 - RIP_760 | 2.26 | -0.09 | 0.01 | 0.39 | 0.10 | 3.63 | 3.65 | 4.23 | 0.00 |
| H | 2018 | BAR_A12 - SLM_001 | 1.83 | 0.52 | 0.16 | 0.00 | 0.11 | 0.79 | 1.54 | 1.93 | 0.00 |
| H | 2018 | RIP_720 - RIP_760 | 1.24 | 0.45 | 0.34 | 0.00 | 0.06 | -0.58 | 1.29 | 1.80 | 0.00 |
| H | 2018 | SLM_001 - RIP_720 | -0.50 | 0.37 | 0.10 | 0.00 | 0.10 | 3.43 | 3.62 | 4.10 | 0.00 |
| H | 2018 | SLM_001 - RIP_760 | 1.44 | 0.07 | 0.01 | 0.37 | 0.08 | 2.85 | 3.03 | 3.64 | 0.00 |
| H | 2019 | BAR_A07 - BAR_A12 | 1.44 | 0.69 | 0.55 | 0.00 | 0.06 | 0.09 | 0.97 | 1.40 | 0.11 |
| H | 2019 | BAR_A07 - RIP_720 | 1.45 | 0.27 | 0.14 | 0.00 | 0.06 | 2.21 | 2.60 | 2.96 | 0.00 |
| H | 2019 | BAR_A07 - RIP_760 | 0.29 | 0.31 | 0.18 | 0.00 | 0.06 | 3.18 | 3.28 | 3.70 | 0.00 |
| H | 2019 | BAR_A07 - SLM_001 | 1.55 | 0.21 | 0.10 | 0.00 | 0.06 | 2.41 | 2.78 | 3.14 | 0.00 |
| H | 2019 | BAR_A12 - RIP_720 | 1.13 | 0.79 | 0.55 | 0.00 | 0.07 | -0.09 | 0.97 | 1.40 | 0.89 |
| H | 2019 | BAR_A12 - RIP_760 | 0.34 | 0.30 | 0.16 | 0.00 | 0.06 | 3.09 | 3.21 | 3.60 | 0.00 |
| H | 2019 | BAR_A12 - SLM_001 | 1.22 | 0.28 | 0.15 | 0.00 | 0.06 | 2.32 | 2.61 | 2.95 | 0.00 |
| H | 2019 | RIP_720 - RIP_760 | -0.09 | 0.68 | 0.46 | 0.00 | 0.07 | 0.97 | 1.24 | 1.50 | 0.00 |
| H | 2019 | RIP_720 - SLM_001 | 0.76 | 0.66 | 0.49 | 0.00 | 0.06 | 0.19 | 0.83 | 1.08 | 0.03 |
| H | 2019 | RIP_760 - SLM_001 | 1.70 | 0.49 | 0.28 | 0.00 | 0.07 | -0.77 | 1.22 | 1.54 | 0.00 |
| H | 2020 | BAR_A07 - BAR_A12 | 0.72 | 0.77 | 0.59 | 0.00 | 0.06 | 0.44 | 0.96 | 1.28 | 0.00 |
| H | 2020 | BAR_A07 - RIP_720 | 1.81 | -0.14 | 0.01 | 0.19 | 0.10 | 3.93 | 4.20 | 4.85 | 0.00 |
| H | 2020 | BAR_A07 - RIP_760 | 0.46 | -0.13 | 0.03 | 0.06 | 0.07 | 5.24 | 5.24 | 5.76 | 0.00 |
| H | 2020 | BAR_A07 - SLM_001 | 2.20 | 0.71 | 0.40 | 0.00 | 0.08 | -0.76 | 1.36 | 1.79 | 0.00 |
| H | 2020 | BAR_A12 - RIP_720 | 1.07 | 0.01 | 0.00 | 0.92 | 0.10 | 3.49 | 3.68 | 4.39 | 0.00 |
| H | 2020 | BAR_A12 - RIP_760 | -0.05 | -0.03 | 0.00 | 0.67 | 0.07 | 4.79 | 4.80 | 5.30 | 0.00 |
| H | 2020 | BAR_A12 - SLM_001 | 2.89 | 0.63 | 0.32 | 0.00 | 0.08 | -1.20 | 1.64 | 2.14 | 0.00 |
| H | 2020 | RIP_720 - RIP_760 | -0.83 | 0.58 | 0.73 | 0.00 | 0.03 | 1.30 | 1.40 | 1.71 | 0.00 |
| H | 2020 | RIP_720 - SLM_001 | 5.65 | 0.14 | 0.02 | 0.11 | 0.09 | -4.69 | 4.70 | 5.37 | 0.00 |
| H | 2020 | RIP_760 - SLM_001 | 5.80 | -0.01 | 0.00 | 0.94 | 0.13 | -5.99 | 5.99 | 6.46 | 0.00 |
| Rn | 2018 | BAR_A12 - RIP720 | 0.28 | 0.17 | 0.01 | 0.22 | 0.14 | 4.22 | 4.26 | 4.82 | 0.00 |
| Rn | 2018 | BAR_A12 - RIP_760 | 2.26 | -0.09 | 0.01 | 0.39 | 0.10 | 3.63 | 3.65 | 4.23 | 0.00 |
| Rn | 2018 | BAR_A12 - SLM_001 | 1.83 | 0.52 | 0.16 | 0.00 | 0.11 | 0.79 | 1.54 | 1.93 | 0.00 |
| Rn | 2018 | RIP_720 - RIP_760 | 1.24 | 0.45 | 0.34 | 0.00 | 0.06 | -0.58 | 1.29 | 1.80 | 0.00 |
| Rn | 2018 | SLM_001 - RIP_720 | -0.50 | 0.37 | 0.10 | 0.00 | 0.10 | 3.43 | 3.62 | 4.10 | 0.00 |
| Rn | 2018 | SLM_001 - RIP_760 | 1.44 | 0.07 | 0.01 | 0.37 | 0.08 | 2.85 | 3.03 | 3.64 | 0.00 |
| Rn | 2019 | BAR_A07 - BAR_A12 | 1.44 | 0.69 | 0.55 | 0.00 | 0.06 | 0.09 | 0.97 | 1.40 | 0.11 |
| Rn | 2019 | BAR_A07 - RIP_720 | 1.45 | 0.27 | 0.14 | 0.00 | 0.06 | 2.21 | 2.60 | 2.96 | 0.00 |
| Rn | 2019 | BAR_A07 - RIP_760 | 0.29 | 0.31 | 0.18 | 0.00 | 0.06 | 3.18 | 3.28 | 3.70 | 0.00 |
| Rn | 2019 | BAR_A07 - SLM_001 | 1.55 | 0.21 | 0.10 | 0.00 | 0.06 | 2.41 | 2.78 | 3.14 | 0.00 |
| Rn | 2019 | BAR_A12 - RIP_720 | 1.46 | 0.27 | 0.13 | 0.00 | 0.06 | 2.12 | 2.44 | 2.85 | 0.00 |
| Rn | 2019 | BAR_A12 - RIP_760 | 0.34 | 0.30 | 0.16 | 0.00 | 0.06 | 3.09 | 3.21 | 3.60 | 0.00 |
| Rn | 2019 | BAR_A12 - SLM_001 | 1.22 | 0.28 | 0.15 | 0.00 | 0.06 | 2.32 | 2.61 | 2.95 | 0.00 |
| Rn | 2019 | RIP_720 - RIP_760 | -0.09 | 0.68 | 0.46 | 0.00 | 0.07 | 0.97 | 1.24 | 1.50 | 0.00 |
| Rn | 2019 | RIP_720 - SLM_001 | 0.76 | 0.66 | 0.49 | 0.00 | 0.06 | 0.19 | 0.83 | 1.08 | 0.03 |
| Rn | 2019 | RIP_760 - SLM_001 | 1.70 | 0.49 | 0.28 | 0.00 | 0.07 | -0.77 | 1.22 | 1.54 | 0.00 |
| Rn | 2020 | BAR_A07 - BAR_A12 | 0.72 | 0.77 | 0.59 | 0.00 | 0.06 | 0.44 | 0.96 | 1.28 | 0.00 |
| Rn | 2020 | BAR_A07 - RIP_720 | 1.81 | -0.14 | 0.01 | 0.19 | 0.10 | 3.93 | 4.20 | 4.85 | 0.00 |
| Rn | 2020 | BAR_A07 - RIP_760 | 0.46 | -0.13 | 0.03 | 0.06 | 0.07 | 5.24 | 5.24 | 5.76 | 0.00 |
| Rn | 2020 | BAR_A07 - SLM_001 | 2.20 | 0.71 | 0.40 | 0.00 | 0.08 | -0.76 | 1.36 | 1.79 | 0.00 |
| Rn | 2020 | BAR_A12 - RIP_720 | 1.07 | 0.01 | 0.00 | 0.92 | 0.10 | 3.49 | 3.68 | 4.39 | 0.00 |
| Rn | 2020 | BAR_A12 - RIP_760 | -0.05 | -0.03 | 0.00 | 0.67 | 0.07 | 4.79 | 4.80 | 5.30 | 0.00 |
| Rn | 2020 | BAR_A12 - SLM_001 | 2.89 | 0.63 | 0.32 | 0.00 | 0.08 | -1.20 | 1.64 | 2.14 | 0.00 |
| Rn | 2020 | RIP_720 - RIP_760 | -0.83 | 0.58 | 0.73 | 0.00 | 0.03 | 1.30 | 1.40 | 1.71 | 0.00 |
| Rn | 2020 | RIP_720 - SLM_001 | 5.65 | 0.14 | 0.02 | 0.11 | 0.09 | -4.69 | 4.70 | 5.37 | 0.00 |
| Rn | 2020 | RIP_760 - SLM_001 | 5.80 | -0.01 | 0.00 | 0.94 | 0.13 | -5.99 | 5.99 | 6.46 | 0.00 |
| G | 2018 | BAR_A12 - RIP720 | 0.13 | 0.46 | 0.17 | 0.00 | 0.09 | 0.17 | 0.28 | 0.34 | 0.00 |
| G | 2018 | BAR_A12 - RIP_760 | 0.07 | 0.36 | 0.13 | 0.00 | 0.09 | 0.28 | 0.34 | 0.41 | 0.00 |
| G | 2018 | BAR_A12 - SLM_001 | -0.07 | 0.46 | 0.25 | 0.00 | 0.07 | 0.37 | 0.39 | 0.44 | 0.00 |
| G | 2018 | RIP_720 - RIP_760 | 0.01 | 0.66 | 0.48 | 0.00 | 0.06 | 0.12 | 0.20 | 0.25 | 0.00 |
| G | 2018 | SLM_001 - RIP_720 | 0.04 | 0.37 | 0.21 | 0.00 | 0.07 | 0.20 | 0.27 | 0.33 | 0.00 |
| G | 2018 | SLM_001 - RIP_760 | 0.08 | 0.40 | 0.20 | 0.00 | 0.07 | 0.08 | 0.22 | 0.28 | 0.00 |
| G | 2019 | BAR_A07 - BAR_A12 | 0.12 | 0.81 | 0.64 | 0.00 | 0.06 | -0.01 | 0.11 | 0.15 | 0.19 |
| G | 2019 | BAR_A07 - RIP_720 | -0.04 | 0.10 | 0.09 | 0.00 | 0.03 | 0.53 | 0.53 | 0.58 | 0.00 |
| G | 2019 | BAR_A07 - RIP_760 | 0.24 | 0.40 | 0.07 | 0.00 | 0.14 | 0.08 | 0.27 | 0.39 | 0.19 |
| G | 2019 | BAR_A07 - SLM_001 | 0.41 | 0.51 | 0.17 | 0.00 | 0.10 | -0.14 | 0.25 | 0.32 | 0.00 |
| G | 2019 | BAR_A12 - RIP_720 | -0.03 | 0.09 | 0.07 | 0.00 | 0.03 | 0.55 | 0.55 | 0.59 | 0.00 |
| G | 2019 | BAR_A12 - RIP_760 | 0.21 | 0.45 | 0.08 | 0.00 | 0.14 | 0.10 | 0.27 | 0.39 | 0.04 |
| G | 2019 | BAR_A12 - SLM_001 | 0.53 | 0.26 | 0.04 | 0.02 | 0.11 | -0.11 | 0.29 | 0.35 | 0.00 |
| G | 2019 | RIP_720 - RIP_760 | 0.44 | 0.97 | 0.04 | 0.03 | 0.44 | -0.44 | 0.53 | 0.58 | 0.00 |
| G | 2019 | RIP_720 - SLM_001 | 0.68 | 0.50 | 0.02 | 0.15 | 0.35 | -0.68 | 0.68 | 0.74 | 0.00 |
| G | 2019 | RIP_760 - SLM_001 | 0.74 | -0.10 | 0.02 | 0.16 | 0.07 | -0.23 | 0.36 | 0.55 | 0.00 |
| G | 2020 | BAR_A07 - BAR_A12 | 0.05 | 0.82 | 0.60 | 0.00 | 0.06 | 0.03 | 0.13 | 0.19 | 0.00 |
| G | 2020 | BAR_A07 - RIP_720 | 0.07 | 0.64 | 0.32 | 0.00 | 0.08 | 0.07 | 0.22 | 0.28 | 0.00 |
| G | 2020 | BAR_A07 - RIP_760 | 0.27 | 0.38 | 0.19 | 0.00 | 0.07 | -0.02 | 0.22 | 0.27 | 0.10 |
| G | 2020 | BAR_A07 - SLM_001 | -0.04 | 0.84 | 0.23 | 0.00 | 0.14 | 0.10 | 0.35 | 0.42 | 0.00 |
| G | 2020 | BAR_A12 - RIP_720 | 0.16 | 0.46 | 0.18 | 0.00 | 0.09 | 0.05 | 0.24 | 0.32 | 0.10 |
| G | 2020 | BAR_A12 - RIP_760 | 0.31 | 0.30 | 0.12 | 0.00 | 0.07 | -0.04 | 0.24 | 0.30 | 0.01 |
| G | 2020 | BAR_A12 - SLM_001 | -0.09 | 1.06 | 0.39 | 0.00 | 0.12 | 0.07 | 0.30 | 0.37 | 0.01 |
| G | 2020 | RIP_720 - RIP_760 | 0.23 | 0.59 | 0.56 | 0.00 | 0.05 | -0.09 | 0.17 | 0.22 | 0.00 |
| G | 2020 | RIP_720 - SLM_001 | 0.02 | 0.88 | 0.31 | 0.00 | 0.12 | 0.02 | 0.30 | 0.39 | 0.04 |
| G | 2020 | RIP_760 - SLM_001 | -0.02 | 0.75 | 0.15 | 0.00 | 0.17 | 0.12 | 0.37 | 0.45 | 0.00 |
| Tair | 2018 | BAR_A12 - RIP720 | 4.78 | 0.95 | 0.54 | 0.00 | 0.08 | -3.85 | 3.93 | 4.44 | 0.00 |
| Tair | 2018 | BAR_A12 - RIP_760 | 3.94 | 1.00 | 0.56 | 0.00 | 0.08 | -3.99 | 4.05 | 4.59 | 0.00 |
| Tair | 2018 | BAR_A12 - SLM_001 | 2.69 | 0.90 | 0.65 | 0.00 | 0.06 | -0.89 | 1.58 | 1.93 | 0.00 |
| Tair | 2018 | RIP_720 - RIP_760 | -0.43 | 1.03 | 0.97 | 0.00 | 0.02 | -0.14 | 0.35 | 0.62 | 0.00 |
| Tair | 2018 | SLM_001 - RIP_720 | 1.39 | 0.81 | 0.86 | 0.00 | 0.03 | 2.96 | 2.96 | 3.20 | 0.00 |
| Tair | 2018 | SLM_001 - RIP_760 | 1.73 | 0.79 | 0.89 | 0.00 | 0.03 | 3.10 | 3.10 | 3.33 | 0.00 |
| Tair | 2019 | BAR_A07 - BAR_A12 | -1.43 | 0.94 | 0.87 | 0.00 | 0.03 | 2.73 | 2.81 | 3.01 | 0.00 |
| Tair | 2019 | BAR_A07 - RIP_720 | 2.05 | 0.94 | 0.66 | 0.00 | 0.06 | -0.72 | 1.99 | 2.50 | 0.00 |
| Tair | 2019 | BAR_A07 - RIP_760 | 10.82 | 0.57 | 0.41 | 0.00 | 0.07 | -1.04 | 2.05 | 2.77 | 0.00 |
| Tair | 2019 | BAR_A07 - SLM_001 | 3.31 | 0.77 | 0.71 | 0.00 | 0.04 | 1.83 | 2.16 | 2.64 | 0.00 |
| Tair | 2019 | BAR_A12 - RIP_720 | 4.82 | 0.93 | 0.65 | 0.00 | 0.06 | -3.44 | 3.70 | 4.21 | 0.00 |
| Tair | 2019 | BAR_A12 - RIP_760 | 12.60 | 0.55 | 0.42 | 0.00 | 0.07 | -3.77 | 4.01 | 4.57 | 0.00 |
| Tair | 2019 | BAR_A12 - SLM_001 | 5.55 | 0.76 | 0.70 | 0.00 | 0.04 | -0.90 | 1.69 | 2.13 | 0.00 |
| Tair | 2019 | RIP_720 - RIP_760 | 5.06 | 0.79 | 0.79 | 0.00 | 0.04 | -0.07 | 0.66 | 1.46 | 0.01 |
| Tair | 2019 | RIP_720 - SLM_001 | 3.79 | 0.72 | 0.84 | 0.00 | 0.03 | 2.55 | 2.72 | 3.07 | 0.00 |
| Tair | 2019 | RIP_760 - SLM_001 | 1.83 | 0.80 | 0.64 | 0.00 | 0.06 | 2.83 | 2.93 | 3.35 | 0.00 |
| Tair | 2020 | BAR_A07 - BAR_A12 | -0.82 | 0.90 | 0.85 | 0.00 | 0.03 | 3.18 | 3.18 | 3.43 | 0.00 |
| Tair | 2020 | BAR_A07 - RIP_720 | 8.54 | 0.65 | 0.47 | 0.00 | 0.06 | -0.59 | 2.15 | 2.61 | 0.01 |
| Tair | 2020 | BAR_A07 - RIP_760 | 8.10 | 0.66 | 0.49 | 0.00 | 0.06 | -0.27 | 2.04 | 2.47 | 0.10 |
| Tair | 2020 | BAR_A07 - SLM_001 | 3.75 | 0.76 | 0.70 | 0.00 | 0.05 | 1.62 | 2.01 | 2.44 | 0.00 |
| Tair | 2020 | BAR_A12 - RIP_720 | 10.85 | 0.64 | 0.42 | 0.00 | 0.07 | -3.71 | 3.92 | 4.56 | 0.00 |
| Tair | 2020 | BAR_A12 - RIP_760 | 10.36 | 0.64 | 0.45 | 0.00 | 0.06 | -3.39 | 3.65 | 4.24 | 0.00 |
| Tair | 2020 | BAR_A12 - SLM_001 | 6.26 | 0.76 | 0.65 | 0.00 | 0.05 | -1.52 | 1.95 | 2.47 | 0.00 |
| Tair | 2020 | RIP_720 - RIP_760 | 0.32 | 0.97 | 0.98 | 0.00 | 0.01 | 0.32 | 0.41 | 0.54 | 0.00 |
| Tair | 2020 | RIP_720 - SLM_001 | 1.19 | 0.85 | 0.79 | 0.00 | 0.04 | 2.21 | 2.30 | 2.64 | 0.00 |
| Tair | 2020 | RIP_760 - SLM_001 | 0.84 | 0.88 | 0.81 | 0.00 | 0.04 | 1.89 | 2.00 | 2.32 | 0.00 |
| VPD | 2018 | BAR_A12 - RIP720 | 0.93 | 0.59 | 0.32 | 0.00 | 0.08 | -0.51 | 0.56 | 0.66 | 0.00 |
| VPD | 2018 | BAR_A12 - RIP_760 | 0.90 | 0.61 | 0.37 | 0.00 | 0.07 | -0.51 | 0.54 | 0.64 | 0.00 |
| VPD | 2018 | BAR_A12 - SLM_001 | 0.76 | 0.63 | 0.35 | 0.00 | 0.08 | -0.38 | 0.47 | 0.57 | 0.00 |
| VPD | 2018 | RIP_720 - RIP_760 | 0.07 | 0.95 | 0.95 | 0.00 | 0.02 | 0.01 | 0.07 | 0.11 | 0.12 |
| VPD | 2018 | SLM_001 - RIP_720 | 0.01 | 0.91 | 0.78 | 0.00 | 0.04 | 0.13 | 0.21 | 0.26 | 0.00 |
| VPD | 2018 | SLM_001 - RIP_760 | -0.02 | 0.93 | 0.78 | 0.00 | 0.04 | 0.12 | 0.21 | 0.25 | 0.00 |
| VPD | 2019 | BAR_A07 - BAR_A12 | 0.02 | 0.87 | 0.99 | 0.00 | 0.01 | 0.13 | 0.13 | 0.16 | 0.00 |
| VPD | 2019 | BAR_A07 - RIP_720 | 0.69 | 0.65 | 0.51 | 0.00 | 0.06 | -0.26 | 0.42 | 0.51 | 0.00 |
| VPD | 2019 | BAR_A07 - RIP_760 | 1.12 | 0.38 | 0.36 | 0.00 | 0.05 | -0.33 | 0.46 | 0.56 | 0.00 |
| VPD | 2019 | BAR_A07 - SLM_001 | 0.47 | 0.74 | 0.69 | 0.00 | 0.05 | -0.15 | 0.29 | 0.37 | 0.00 |
| VPD | 2019 | BAR_A12 - RIP_720 | 0.67 | 0.74 | 0.51 | 0.00 | 0.07 | -0.39 | 0.49 | 0.56 | 0.00 |
| VPD | 2019 | BAR_A12 - RIP_760 | 1.11 | 0.43 | 0.37 | 0.00 | 0.06 | -0.47 | 0.54 | 0.61 | 0.00 |
| VPD | 2019 | BAR_A12 - SLM_001 | 0.46 | 0.84 | 0.68 | 0.00 | 0.05 | -0.28 | 0.34 | 0.42 | 0.00 |
| VPD | 2019 | RIP_720 - RIP_760 | 0.52 | 0.67 | 0.76 | 0.00 | 0.04 | 0.02 | 0.14 | 0.23 | 0.00 |
| VPD | 2019 | RIP_720 - SLM_001 | 0.11 | 0.85 | 0.76 | 0.00 | 0.04 | 0.11 | 0.25 | 0.30 | 0.00 |
| VPD | 2019 | RIP_760 - SLM_001 | -0.26 | 1.07 | 0.55 | 0.00 | 0.10 | 0.14 | 0.29 | 0.37 | 0.00 |
| VPD | 2020 | BAR_A07 - BAR_A12 | 0.04 | 0.86 | 0.88 | 0.00 | 0.03 | 0.15 | 0.17 | 0.24 | 0.00 |
| VPD | 2020 | BAR_A07 - RIP_720 | 1.15 | 0.31 | 0.21 | 0.00 | 0.06 | -0.25 | 0.49 | 0.57 | 0.00 |
| VPD | 2020 | BAR_A07 - RIP_760 | 1.05 | 0.32 | 0.32 | 0.00 | 0.04 | -0.15 | 0.39 | 0.48 | 0.00 |
| VPD | 2020 | BAR_A07 - SLM_001 | 0.89 | 0.62 | 0.51 | 0.00 | 0.06 | -0.39 | 0.47 | 0.56 | 0.00 |
| VPD | 2020 | BAR_A12 - RIP_720 | 1.11 | 0.39 | 0.26 | 0.00 | 0.06 | -0.39 | 0.52 | 0.60 | 0.00 |
| VPD | 2020 | BAR_A12 - RIP_760 | 1.06 | 0.35 | 0.33 | 0.00 | 0.05 | -0.30 | 0.44 | 0.51 | 0.00 |
| VPD | 2020 | BAR_A12 - SLM_001 | 0.91 | 0.68 | 0.51 | 0.00 | 0.06 | -0.53 | 0.57 | 0.65 | 0.00 |
| VPD | 2020 | RIP_720 - RIP_760 | 0.32 | 0.73 | 0.83 | 0.00 | 0.03 | 0.10 | 0.15 | 0.19 | 0.00 |
| VPD | 2020 | RIP_720 - SLM_001 | 0.40 | 0.83 | 0.44 | 0.00 | 0.08 | -0.13 | 0.27 | 0.39 | 0.00 |
| VPD | 2020 | RIP_760 - SLM_001 | -0.05 | 1.19 | 0.58 | 0.00 | 0.09 | -0.23 | 0.29 | 0.40 | 0.00 |

1. http://www.prism.oregonstate.edu/documents/PRISM_datasets.pdf [↑](#footnote-ref-1)
